# K_2P_18.1 translates T cell receptor signals into thymic regulatory T cell development

**DOI:** 10.1038/s41422-021-00580-z

**Published:** 2021-10-26

**Authors:** Tobias Ruck, Stefanie Bock, Steffen Pfeuffer, Christina B. Schroeter, Derya Cengiz, Paul Marciniak, Maren Lindner, Alexander Herrmann, Marie Liebmann, Stjepana Kovac, Lukas Gola, Leoni Rolfes, Marc Pawlitzki, Nils Opel, Tim Hahn, Udo Dannlowski, Thomas Pap, Felix Luessi, Julian A. Schreiber, Bernhard Wünsch, Tanja Kuhlmann, Guiscard Seebohm, Björn Tackenberg, Patricia Seja, Frank Döring, Erhard Wischmeyer, Achmet Imam Chasan, Johannes Roth, Luisa Klotz, Gerd Meyer zu Hörste, Heinz Wiendl, Tobias Marschall, Stefan Floess, Jochen Huehn, Thomas Budde, Tobias Bopp, Stefan Bittner, Sven G. Meuth

**Affiliations:** 1 Department of Neurology, Medical Faculty, Heinrich-Heine-University, Düsseldorf, Germany; 2 Department of Neurology with Institute of Translational Neurology, University of Münster, Münster, Germany; 3 Institute for Translational Psychiatry, University of Münster, Münster, Germany; 4 Institute of Experimental Musculoskeletal Medicine (IMM), University of Münster, Münster, Germany; 5 Department of Neurology, Focus Program Translational Neuroscience (FTN) and Immunotherapy (FZI), Rhine Main Neuroscience Network (rmn2), University Medical Center of the Johannes Gutenberg University Mainz, Mainz, Germany; 6 Institute of Pharmaceutical and Medicinal Chemistry, University of Münster, Münster, Germany; 7 Cellular Electrophysiology and Molecular Biology, Institute for Genetics of Heart Diseases (IfGH), University of Münster, Münster, Germany; 8 Institute of Neuropathology, University of Münster, Münster, Germany; 9 Department of Neurology, Philipps-University, Marburg, Germany; 10 Laboratory of Neurobiology, University of Helsinki, Helsinki, Finland; 11 Molecular Electrophysiology, Institute of Physiology and Center of Mental Health, University of Würzburg, Würzburg, Germany; 12 Institute of Immunology, University of Münster, Münster, Germany; 13 Institute for Medical Biometry and Bioinformatics, Medical Faculty, Heinrich Heine University, Düsseldorf, Germany; 14 Department of Experimental Immunology, Helmholtz Centre for Infection Research, Braunschweig, Germany; 15 Institute for Physiology I, University of Münster, Münster, Germany; 16 Institute of Immunology, Focus Program Immunotherapy (FZI), University Medical Center of the Johannes Gutenberg University Mainz, Mainz, Germany

**Keywords:** Calcium signalling, Autoimmunity, Ion channel signalling

## Abstract

It remains largely unclear how thymocytes translate relative differences in T cell receptor (TCR) signal strength into distinct developmental programs that drive the cell fate decisions towards conventional (Tconv) or regulatory T cells (Treg). Following TCR activation, intracellular calcium (Ca^2+^) is the most important second messenger, for which the potassium channel K_2P_18.1 is a relevant regulator. Here, we identify K_2P_18.1 as a central translator of the TCR signal into the thymus-derived Treg (tTreg) selection process. TCR signal was coupled to NF-κB-mediated K_2P_18.1 upregulation in tTreg progenitors. K_2P_18.1 provided the driving force for sustained Ca^2+^ influx that facilitated NF-κB- and NFAT-dependent expression of FoxP3, the master transcription factor for Treg development and function. Loss of K_2P_18.1 ion-current function induced a mild lymphoproliferative phenotype in mice, with reduced Treg numbers that led to aggravated experimental autoimmune encephalomyelitis, while a gain-of-function mutation in K_2P_18.1 resulted in increased Treg numbers in mice. Our findings in human thymus, recent thymic emigrants and multiple sclerosis patients with a dominant-negative missense K_2P_18.1 variant that is associated with poor clinical outcomes indicate that K_2P_18.1 also plays a role in human Treg development. Pharmacological modulation of K_2P_18.1 specifically modulated Treg numbers in vitro and in vivo. Finally, we identified nitroxoline as a K_2P_18.1 activator that led to rapid and reversible Treg increase in patients with urinary tract infections. Conclusively, our findings reveal how K_2P_18.1 translates TCR signals into thymic T cell fate decisions and Treg development, and provide a basis for the therapeutic utilization of Treg in several human disorders.

## Introduction

Regulatory T cells (Tregs) are essential mediators of immune tolerance. Alterations in their numbers or function contribute to the pathogenesis of autoimmunity and are central in tumor evasion mechanisms in malignant disorders.^1–4^

Thymic Treg (tTreg) and conventional T cells (Tconv) both differentiate from thymocytes in response to T cell receptor (TCR) signaling. tTreg receive a stronger TCR signal than Tconv during thymocyte development,^5^ and this is essential for the induction of tTreg-specific epigenetic changes and gene expression patterns.^6^ A sufficiently strong TCR signal results in the expression of proximal IL-2 signaling components, facilitating cytokine-mediated induction of FoxP3, the master transcription factor for Treg development and distinctive feature of Treg.^7,8^ Data from TCR-transgenic mouse studies suggest that TCR signal strength required for tTreg development lies between the signal strengths driving positive and negative selection.^9,10^

The induction and stabilization of FoxP3 expression are critical to tTreg fate, and are tightly regulated by a variety of signaling pathways such as NF-κB, PI3K/Akt, STAT5 and NFAT.^11^ Given that a single thymocyte–antigen presenting cell (APC) encounter is believed to determine tTreg differentiation,^11^ the regulation of these signaling pathways must be highly efficient and dependent on the integration of various cofactors, although only a few are known so far.^12^ One well-characterized factor that affects this process is intracellular Ca^2+^, which is known to regulate NFAT and NF-κB signaling.^13^ Therefore, an enhanced Ca^2+^ signal in response to TCR activation might be decisive for tTreg fate, as there is intraclonal competition for a restricted resource (self-antigens).^14^

Interestingly, previous studies have demonstrated that store-operated Ca^2+^ entry (SOCE) via Ca^2+^ release-activated Ca^2+^ (CRAC) channels is essential for Treg development and function. T cell-specific ablation of stromal interaction molecule 1 and 2 (STIM1 and STIM2), both of which are CRAC channel calcium-sensing proteins, led to a lymphoproliferative phenotype and a selective decrease in Treg numbers in the thymus and lymphoid organs in mice.^15^ However, pharmacological CRAC channel inhibitors only affected peripherally-induced Treg (pTreg).^16^ Therefore, CRAC channels are presumably not the major determinant of Ca^2+^-mediated regulation of tTreg development. Other so far unidentified factors should be involved in the regulation of Ca^2+^ dynamics related to tTreg development. As potassium (K^+^) channels are the driving force for sustained Ca^2+^ influx by mediating a hyperpolarizing outward current, they represent likely candidates that are involved in these processes.^17,18^ In the family of K^+^ channels, two-pore domain K^+^ (K_2P_) channels are especially suited to serve as such a regulatory factor for tTreg development as they are major determinants of K^+^ conductance, adjust excitability and counteract membrane potential depolarization. Moreover, K_2P_ channels are regulated by various extra- and intracellular stimuli, thereby integrating diverse signals in multiple circumstances ranging from basic cellular functions to complex pathogenic processes.^19^ In accordance with their complex and variable functions, we and others have identified an essential role for K_2P_ channels in (auto)immune processes such as T cell activation, modulation of effector functions and regulation of immune cell trafficking across the blood–brain barrier.^20–23^

Given that the K_2P_ channel K_2P_18.1 (also known as TWIK-related spinal cord K^+^ channel, TRESK; encoded by the *Kcnk18* gene) is strongly expressed in the thymus and spleen (as well as the spinal cord and brain),^24,25^ we speculated that it may play a role in tTreg development and function. Two unique features of K_2P_18.1 specifically argue for this potential: (1) Ca^2+^ influx induces calmodulin/calcineurin-mediated dephosphorylation of intracellular K_2P_18.1 domains, leading to an increased probability of the channel being open,^26^ and (2) sustained Ca^2+^ influx also induces calmodulin/calcineurin-mediated activation of the NFAT pathway, which is essential for T cell development.^27^ Interestingly, the binding affinity of the intracellular domain of K_2P_18.1 (calcineurin binding motif: PQIVID in mouse and PQIIIS in human K_2P_18.1) to calcineurin considerably exceeds that of NFAT1 (PxIxIT motif), and is the highest among all natural proteins identified so far.^28^

Therefore, in this study we sought to determine whether K_2P_18.1 plays a role in translating TCR signal strength to induce thymocyte differentiation into Treg. We show that TCR signal strength is coupled to K_2P_18.1 expression in tTreg progenitors. Via modulation of intracellular Ca^2+^ signals, K_2P_18.1 facilitates NF-κB- and NFAT-mediated FoxP3 expression, and thereby tTreg development and maturation. Pharmacological modulation of K_2P_18.1 allows to rapidly and reversibly adjust Treg numbers.

## Results

### Lymphoproliferative phenotype and reduced Treg numbers in *Kcnk18*^***−/−***^ mice

**Fig. 1:**
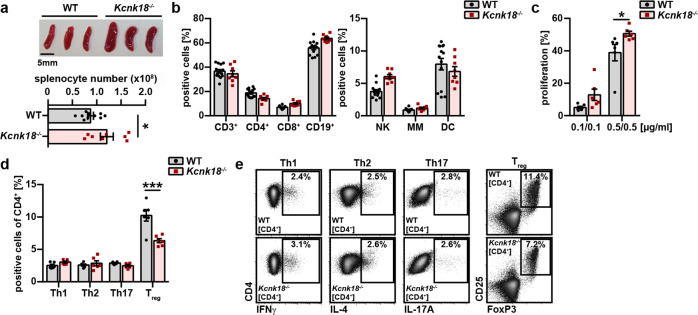
*Kcnk18*^−***/−***^ mice show a lymphoproliferative phenotype due to reduced Treg numbers. **a** Upper panel: photographs of spleens from WT littermate and *Kcnk18*^−/−^ mice. Scale bar, 5 mm. Lower panel, absolute numbers of splenocytes in WT and *Kcnk18*^−/−^ mice (*n* = 8). **b** Relative proportions for major immune cell subsets of splenocytes from WT and *Kcnk18*^−/−^ mice. NK natural killer cells, MM monocytes and macrophages, DC dendritic cells (*n* = 5 per group). **c** CFSE (carboxyfluorescein succinimidyl ester) proliferation assay of splenocytes isolated from WT and *Kcnk18*^−/−^ mice. Splenocytes were stimulated with plate-bound anti-CD3 and soluble anti-CD28 antibodies at the indicated concentrations for 72 h and analyzed by flow cytometry (*n* = 6). **d** Frequency of Th1 (CD4^+^FoxP3^–^IFNγ^+^), Th2 (CD4^+^FoxP3^–^IL4^+^), Th17 (CD4^+^FoxP3^–^IL17^+^) and CD4^+^CD25^+^FoxP3^+^ Tregs of splenic CD4^+^ T cells (*n* = 6). **e** Representative flow cytometry plots of Th1, Th2, Th17 and Treg gating. Data are represented as means ± SEM. *P < 0.05; **P < 0.01; ***P < 0.001.

Ca^2+^ signaling pathways are essential for T cell development and function and in silico models predicted a critical role for K_2P_18.1 in these processes.^17,29^ Therefore, we investigated the immunological consequences of genetic *Kcnk18* deletion (Supplementary information, Fig. S1a). *Kcnk18*^***−/−***^ mice showed healthy development by histological analyses of central nervous system (CNS) and other organs (data not shown). However, *Kcnk18* deletion led to a mild lymphoproliferative phenotype with enlarged spleens and higher absolute cell numbers (Fig. 1a), whereas relative proportions of immune cell subsets were not changed (Fig. 1b). Under TCR stimulation, splenocytes from *Kcnk18*^***−/−***^ mice showed augmented proliferation compared to C57BL/6 wild-type (WT) mice (Fig. 1c), potentially related to enhanced immune cell activation and/or insufficient suppression by regulatory immune cells. However, under naïve conditions, CD4^+^ and CD8^+^ T cells displayed a similar activation status in WT and *Kcnk18*^***−/−***^ mice (Supplementary information, Fig. S2a). A more detailed investigation of T helper (Th) subsets revealed reduced frequencies of CD4^+^CD25^+^FoxP3^+^ Treg in the *Kcnk18*^***−/−***^ spleens compared to WT ones, whereas frequencies of Th1, Th2 and Th17 subsets were similar (Fig. 1d, e). In further support of a Treg-specific phenotype, in vitro induction of Th1, Th2 and Th17 subsets was unaltered in *Kcnk18*^−^^***/−***^ mice (Supplementary information, Fig. S2b). Thus, loss of K_2P_18.1 function is associated with reduced Treg numbers.

### *Kcnk18*^*−/−*^ mice show a defect in thymus-derived Treg development

**Fig. 2:**
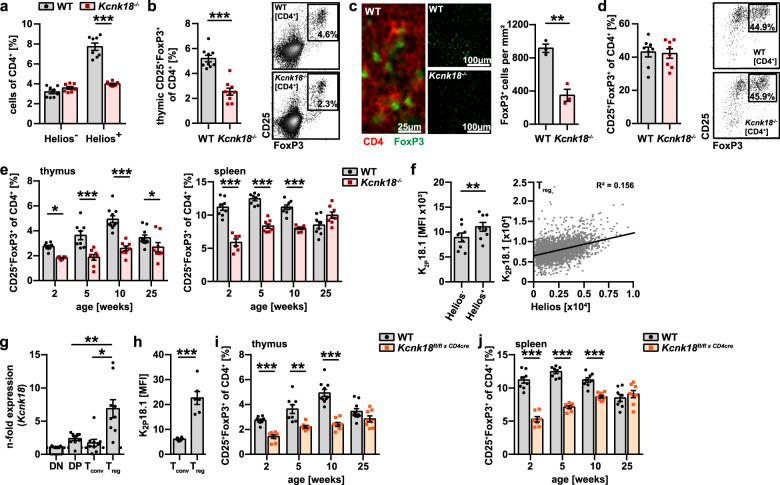
Impaired tTreg development in the thymus leads to reduced Treg numbers in *Kcnk18*^−*/*−^ mice. **a** Frequency of Helios-positive and -negative WT and *Kcnk18*^−*/*−^ Tregs in CD4^+^ T cells from spleen evaluated by flow cytometry (*n* = 8). **b** Frequency of Treg in thymic single positive (SP) CD4 thymocytes. Left, bar graphs; right, representative dot plots (*n* = 8). **c** Immunofluorescence staining for FoxP3 (green) and CD4 (red) in WT and *Kcnk18*^−*/*−^ thymus. Scale bars represent 25 µm or 100 µm, respectively. Left panel, representative staining; right panel, quantification of CD4^+^FoxP3^+^ cells/mm^2^ (*n* = 3). **d** In vitro induced Treg (iTreg): CD4^+^CD25^−^ naïve T cells were isolated from spleens by magnetic-activated cell sorting (MACS), stimulated with plate-bound anti-CD3 (1 µg/mL) and soluble anti-CD28 (2 μg/mL) in the presence of TGFβ (5 ng/mL) for 3 days, and then analyzed by flow cytometry. Left, bar graphs; right, representative dot plots (*n* = 8). **e** Age-dependent frequency of WT and *Kcnk18*^−*/*−^ Tregs in thymus (left) and spleen (right) (*n* = 8). **f** Left: K_2P_18.1 expression in Helios-positive and -negative WT Tregs isolated from spleen; right: correlation of K_2P_18.1 and Helios expression (*n* = 8). **g** qPCR for *Kcnk18* expression in double negative (DN), double positive (DP), CD4 SP (Tconv) and Treg isolated from WT thymus. Relative *Kcnk18* expression (2^–∆∆CT^) compared to DN is depicted (*n* = 8). **h** Flow cytometry for K_2P_18.1 expression in Tconv (CD4^+^CD25^−^FoxP3^−^) and Treg (CD4^+^CD25^+^FoxP3^+^) isolated from WT thymus (*n* = 6). **i**, **j** Age-dependent frequency of Treg from WT and *Kcnk18*^*fl/fl × CD4cre*^ thymus (**i**) and spleen (**j**) evaluated by flow cytometry (*n* = 8). Data are represented as means ± SEM. *P < 0.05; **P < 0.01; ***P < 0.001.

Next, we asked whether the reduction of Treg in *Kcnk18*^***−/−***^ mice is a general effect observed in the whole Treg compartment or confined to thymus-derived (tTreg) or peripherally-induced Treg (pTreg). The observation that only tTreg (Helios-positive) but not pTreg (Helios-negative) were reduced (Fig. 2a) points toward a tTreg defect. In support of this hypothesis, (1) we observed significantly reduced Treg numbers in the thymus (tTreg, Fig. 2b, c); (2) in vitro generation of Treg from naïve cells was similar in Kcnk18^−/−^ and WT mice (Fig. 2d); and (3) tTreg reduction was already evident at early developmental stages and was compensated by pTreg during aging (Fig. 2e). K_2P_18.1 expression was higher on Helios-positive Treg than Helios-negative Treg and positively correlated with Helios expression (Fig. 2f). Moreover, *Kcnk18* and K_2P_18.1 were higher expressed in tTreg compared to other T cell developmental stages in the thymus at mRNA (Fig. 2g) and protein levels, respectively (Fig. 2h; Supplementary information, Fig. S8a). Thus, high K_2P_18.1 expression is specific for tTreg and functionally relevant to tTreg development. Consistent with this, a CD4^+^ cell-specific *Kcnk18*-knockout mouse exhibited a similar phenotype with an age-dependent reduction of tTreg in thymus (Fig. 2i) and spleen (Fig. 2j). Moreover, we observed no differences in major thymocyte or thymic APC subsets (Supplementary information, Fig. S3a, b). Cumulatively, these data show that Treg reduction observed in *Kcnk18*^***−/−***^ mice is due to a defect in tTreg development.

### K_2P_18.1 deficiency has no impact on Treg phenotype and function

**Fig. 3:**
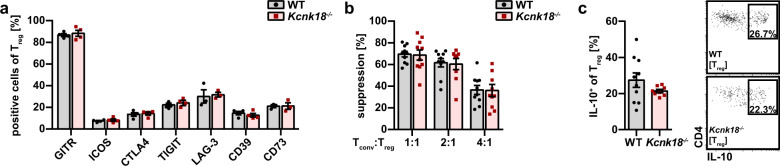
The function of Treg is not altered in *Kcnk18*^−*/*−^ mice. **a** Surface expression of the indicated effector molecules on Tregs isolated from spleens of WT and *Kcnk18*^−*/*−^ mice (*n* = 3–4). **b** Suppression assay: Tconvs (CD4^+^CD25^–^ T cells) were isolated from 2D2 (TCR^MOG^) transgenic mice and Tregs from WT and *Kcnk18*^−*/*−^ mice, and then Tconvs were stained with CFSE and co-cultured with Tregs (WT or *Kcnk18*^−*/*−^, 1:1, 2:1, 4:1) and MOG (myelin oligodendrocyte glycoprotein)-loaded APCs for 3 days. Suppression of Tconv proliferation by Treg was calculated as proportion of proliferated Tconv with Treg divided by proportion of proliferated Tconv without Treg (*n* = 10 per group). **c** Intracellular cytokine staining for IL-10 (left, bar graphs; right, representative dot plots): MACS-isolated splenic CD4^+^ T cells were stimulated with plate-bound anti-CD3 (2.0 μg/mL) and soluble anti-CD28 (4.0 μg/mL) for 24 h, then re-stimulated with leukocyte activation cocktail (0.5 µg/mL, containing PMA, ionomycin and Brefeldin A) for 4 h and analyzed by flow cytometry. CD4^+^ T cells were used for gating on Treg (CD25^+^FoxP3^+^); IL-10 expression in Treg was assessed by plotting IL-10 against CD4 (*n* = 10). Data are represented as means ± SEM. *P < 0.05; **P < 0.01; ***P < 0.001.

So far, we found reduced tTreg numbers, therefore we next asked whether the observed defect in tTreg development also affects Treg phenotype and function. The phenotypes of *Kcnk18*^−*/*−^ and WT Tregs were similar as indicated by the expression levels of various Treg effector molecules (Fig. 3a). Suppression assays of Tconv proliferation by Treg revealed no differences in Treg function (Fig. 3b), which was further substantiated by unchanged Treg-related IL-10 production (Fig. 3c). Thus, loss of K_2P_18.1 function led to reduced tTreg numbers, whereas the Treg function is not impaired.

### tTreg development is regulated by the ion-current function of K_2P_18.1

**Fig. 4:**
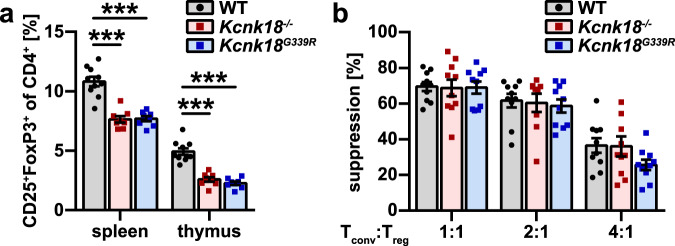
The ion-current function of K_2P_18.1 regulates the generation of tTreg. **a** Treg proportions in thymus and spleen from WT, *Kcnk18*^−*/*−^ and *Kcnk18*^*G339R*^ mice (*n* = 5 per group). **b** Suppression assay of proliferation of Tconv from 2D2 mice by Tregs isolated from WT, *Kcnk18*^−*/*−^ and *Kcnk18*^*G339R*^ mice as described in Fig. 3b (*n* = 10 per group). Data are represented as means ± SEM. *P < 0.05; **P < 0.01; ***P < 0.001.

To determine whether the impaired development of tTreg observed in response to loss of K_2P_18.1 function was related to the ion-current function of K_2P_18.1, we generated a mutant mouse line with a single amino acid change (G339R) eliminating ion-current flow through K_2P_18.1 (Supplementary information, Fig. S1b).^30^ We found the same phenotype with reduced tTreg in spleen and thymus (Fig. 4a) and preserved Treg function (Fig. 4b) in *Kcnk18*^*G339R*^ mice as observed in *Kcnk18*^−*/*−^ mice. Thus, proper tTreg development depends on the ion-current through K_2P_18.1 and not on interactions with other proteins via its intracellular domain.

### K_2P_18.1 regulates central signaling pathways for tTreg development

**Fig. 5:**
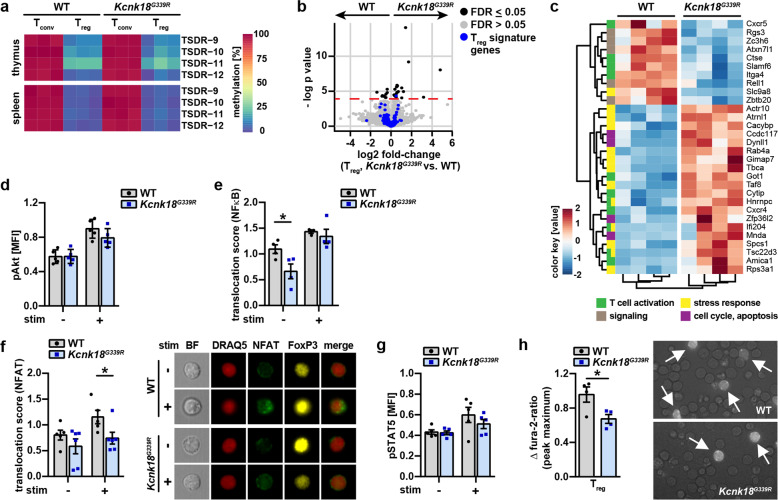
Signaling pathways related to tTreg development are altered in *Kcnk18*^*G339R*^ mice. **a** Methylation status of the FoxP3-TSDR in WT-*Foxp3*^*RFP*^ and *Kcnk18*^*G339R*^*/FoxP3*^*RFP*^ Tregs isolated from thymus or spleen (*n* = 4 per group). **b**, **c** mRNA sequencing of WT-*FoxP3*^*RFP*^ and *Kcnk18*^*G339R*^*/FoxP3*^*RFP*^ Tregs (CD3^+^CD4^+^CD8^–^CD25^+^FoxP3^+^) isolated from thymus: volcano plot of FoxP3-regulated Treg signature genes (**b**) and heatmap of unbiased analysis for significantly differentially expressed genes (FDR < 0.05), automated gene clustering (hclust) and GO-term annotation (*n* = 4 per group) (**c**). **d** Phospho-Akt (pAkt) expression in WT and *Kcnk18*^*G339R*^ tTregs isolated from thymus analyzed by flow cytometry (*n* = 5). **e**, **f** Imaging flow cytometry of NF-κB (**e**) and NFAT (**f**) translocation to the nucleus in WT and *Kcnk18*^*G339R*^ tTregs isolated from thymus. Cells were either left untreated or stimulated (stim) with plate-bound anti-CD3 (2 μg/mL) and soluble anti-CD28 (4 μg/mL) antibodies for 3 h (*n* = 4–5)_._ Translocation scores under naïve and stimulated conditions were calculated. For **f**, right, representative images; BF, bright field; DRAQ5 (nuclear staining, red), NFAT (green), FoxP3 (yellow), merge (DRAQ5, NFAT). **g** Phospho-STAT5 (pSTAT5) expression analyzed by flow cytometry in naïve and stimulated tTregs from WT and *Kcnk18*^*G339R*^ mice (*n* = 5). **h** Live-cell Ca^2+^ imaging of WT-*Foxp3*^*RFP*^ and *Kcnk18*^*G339R*^*/Foxp3*^*RFP*^ tTregs isolated from thymus. Left, peak maximum of ∆ fura-2 ratio; right, representative fluorescence microscopy images showing RFP expression (*n* = 4). Data are represented as means ± SEM. *P < 0.05; **P < 0.01; ***P < 0.001.

Next, we investigated the molecular mechanisms that connect K_2P_18.1 ion-current and the processes of tTreg development. Consistent with a regular function of tTreg, we found similar demethylation of the FoxP3-TSDR (forkhead box P3 Treg-specific demethylated region) in WT and *Kcnk18*^*G339R*^ mice, which corresponds to stability of FoxP3 expression and immunosuppressive function of Treg (Fig. 5a).^31^ Furthermore, WT and *Kcnk18*^*G339R*^ tTregs showed similar expression patterns of FoxP3-regulated tTreg signature genes, indicating that the defect in tTreg development occurs before established FoxP3 expression (Fig. 5b). Unbiased bulk RNA-sequencing revealed that compared to WT tTreg, *Kcnk18*^*G339R*^ tTreg were characterized by gene expression patterns for decreased T cell activation and signaling and increased stress responses, cell cycle and apoptosis (Fig. 5c; Supplementary information, Fig. S4). However, cell cycle (Supplementary information, Fig. S5a), proliferation (Supplementary information, Fig. S5b), apoptosis rates (Supplementary information, Fig. S5c) and thymus egress rates (Supplementary information, Fig. S5d) were comparable between WT and *Kcnk18*^*G339R*^ tTregs. For T cell signaling, pathways involving PI3K/Akt, NF-κB, NFAT and STAT5 have been implicated as important regulators of FoxP3 expression (Supplementary information, S1d). Therefore, we next investigated whether loss of the K_2P_18.1 ion-current leads to alterations in these cascades.^11^ In comparison to WT tTreg, *Kcnk18*^*G339R*^ tTreg showed no relevant alterations in PI3K/Akt signaling (Fig. 5d). In contrast, NF-κB translocation to the nucleus was reduced in *Kcnk18*^*G339R*^ tTreg, even though external TCR stimulation was able to compensate for those differences (Fig. 5e). Similar to NF-κB, we found decreased NFAT translocation to the nucleus in *Kcnk18*^*G339R*^ tTreg (Fig. 5f); however, those alterations were not compensated by external TCR stimulation. K_2P_18.1 might not only influence the first step of TCR-dependent remodeling of the *FoxP3* locus, but also the second cytokine-dependent step inducing FoxP3 expression via IL-2 signaling. However, we detected no differences in phosphorylated STAT5 levels, the major downstream target of IL-2-mediated signaling (Fig. 5g). Thus, loss of K_2P_18.1 ion-current function is associated with reduced nuclear translocation of NF-κB and NFAT, which are both regulated by distinct intracellular Ca^2+^ signals.^32,33^ K_2P_18.1 might therefore regulate NF-κB and NFAT signaling by fine-tuning intracellular Ca^2+^ concentration ([Ca^2+^]_i_). Consistent with this, live-cell Ca^2+^ imaging of tTreg revealed reduced Ca^2+^ influx upon TCR stimulation in cells from *Kcnk18*^*G339R*^ mice as compared to WT mice (Fig. 5h). Therefore, K_2P_18.1 is able to regulate essential signaling pathways upstream of FoxP3 expression via modulation of [Ca^2+^]_i_.

### K_2P_18.1 expression provides a selection advantage for tTreg and is coupled to TCR signal strength via NF-κB signaling

**Fig. 6:**
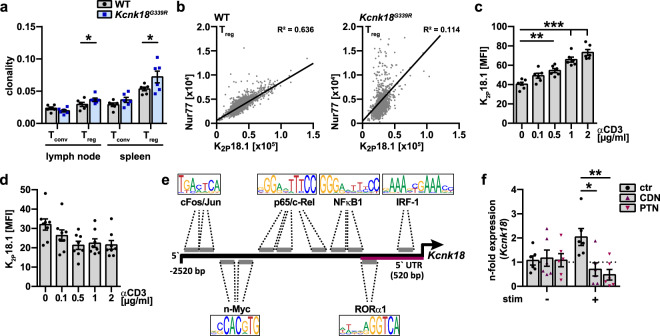
K_2P_18.1 expression is coupled to TCR signaling and provides a specific competitive advantage for tTreg in thymic selection. **a** Clonality of TCR repertoire in WT (*WT-Foxp3*^*RFP*^) and *Kcnk18*^*G339R*^*/Foxp3*^*RFP*^ Tconv and Treg in spleen and lymph nodes evaluated by deep sequencing (*n* = 6). **b** Correlation between K_2P_18.1 and Nur77 expression (area) in tTregs isolated from WT and *Kcnk18*^*G339R*^ thymus. **c**, **d** Flow cytometry analysis for K_2P_18.1 expression in tTreg (**c**) and Tconv (**d**) isolated from thymus and stimulated with the indicated concentrations of anti-CD3 antibody and soluble anti-CD28 (2 µg/mL) (*n* = 6–8 per group). **e** Scheme of the region 2520 bp (5′UTR in magenta) upstream of *Kcnk18* start codon showing putative transcription factor binding sites including NF-κB1 and p65/c-Rel. *Kcnk18* (Entrez gene ID: 332396) was used for transcription factor binding site analysis with ConSite. See Supplementary information, Table S1 for detailed locations of transcription factor binding sites. **f** qPCR for *Kcnk18* expression on WT tTreg isolated from thymus. Naïve or stimulated tTreg (stim, plate-bound anti-CD3 (2 μg/mL) and soluble anti-CD28 (4 μg/mL) antibodies for 3 h) were treated with different inhibitors of NF-κB signaling (20 µM CDN or 5 µM PTN for 3 h) (*n* = 6). Data are represented as means ± SEM. *P < 0.05; **P < 0.01; ***P < 0.001.

For thymic selection, tTregs require strong TCR signals and thus also high [Ca^2+^]_i._^6,34–36^ We therefore asked whether K_2P_18.1 is involved in the TCR-dependent selection process and whether there is a link between TCR signal and K_2P_18.1 to facilitate the Ca^2+^ signal in tTreg. Consistent with a specific role of K_2P_18.1 in the competitive processes of thymic tTreg selection, *Kcnk18*^*G339R*^ mice showed a significantly narrowed Treg TCR repertoire in the periphery, whereas the Tconv TCR repertoire was not altered compared to WT mice (Fig. 6a). tTreg that received a strong TCR signal (as indicated by high Nur77 expression level)^5^ showed high K_2P_18.1 expression level, whereas this correlation was nearly abrogated in *Kcnk18*^*G339R*^ mice (Fig. 6b). This indicates a biological interrelation of TCR signal and K_2P_18.1 via its ion-current function. In agreement, increasing concentrations of an anti-CD3 antibody led to a dose-dependent enhancement of K_2P_18.1 expression in tTreg (Fig. 6c), whereas Tconv showed rather decreasing K_2P_18.1 expression upon stimulation (Fig. 6d). These data indicate a specific link between TCR signal and K_2P_18.1 expression in tTreg. As the *Kcnk18* gene locus shows multiple putative binding sites for the NF-κB-related protein c-Rel and NF-κB1, *Kcnk18* expression might be connected to the TCR signal via NF-κB signaling (Fig. 6e; Supplementary information, Table S1). Consistent with this hypothesis, we observed that upregulation of *Kcnk18* expression under TCR stimulation was inhibited by pharmacological blockade of NF-κB activation using cardamonin (CDN) or parthenolide (PTN) (Fig. 6f).^37,38^ These data collectively demonstrate that K_2P_18.1 expression provides a specific selection advantage for tTreg in the thymus and is coupled to TCR signal strength via NF-κB signaling.

### K_2P_18.1 channel function is important for FoxP3 induction in tTreg progenitors

**Fig. 7:**
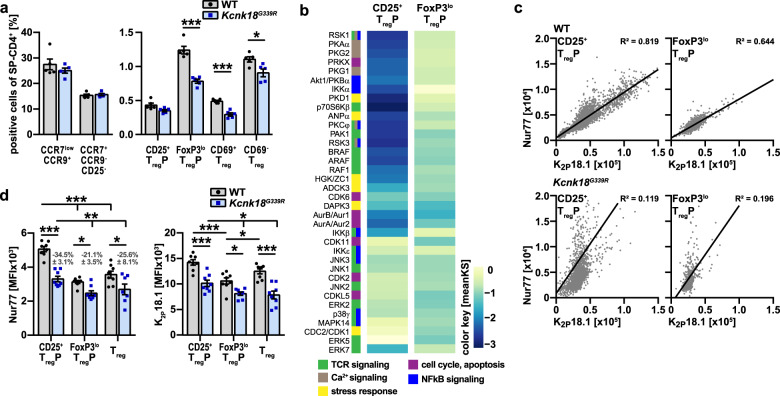
K_2P_18.1 is important for FoxP3 induction in thymic Treg progenitors. **a** Frequencies of the indicated developing tTreg cell populations (including FoxP3^lo^ TregP (CD3^+^CD4^+^CD8^–^CD25^–^FoxP3^lo^) and CD25^+^ TregP (CD3^+^CD4^+^CD8^–^CD25^+^FoxP3^−^)) in the thymus of WT and *Kcnk18*^*G339R*^*/Foxp3*^*RFP*^ mice (*n* = 5). **b** Kinase activity profiles of CD25^+^ TregP and FoxP3^lo^ TregP sorted from *Kcnk18*^*G339R*^*/Foxp3*^*RFP*^ thymus compared to the corresponding WT-*Foxp3*^*RFP*^ cell subsets and annotation of kinases to the indicated signaling pathways. Color key indicates the mean kinase statistic (meanKS) of the differential kinase activity. A positive meanKS indicates enhanced kinase activity in *Kcnk18*^*G339R*^; negative meanKS shows reduced kinase activity in *Kcnk18*^*G339R*^ (*n* = 6). **c** Correlation between K_2P_18.1 and Nur77 expression (area) in ex vivo CD25^+^ TregP and FoxP3^lo^ TregP isolated from WT (upper panel) and *Kcnk18*^*G339R*^ (lower panel) thymus. **d** K_2P_18.1 and Nur77 expression (MFI) in these cell types from WT or *Kcnk18*^*G339R*^ thymus (*n* = 6). Data are represented as means ± SEM. *P < 0.05; **P < 0.01; ***P < 0.001.

**Fig. 8:**
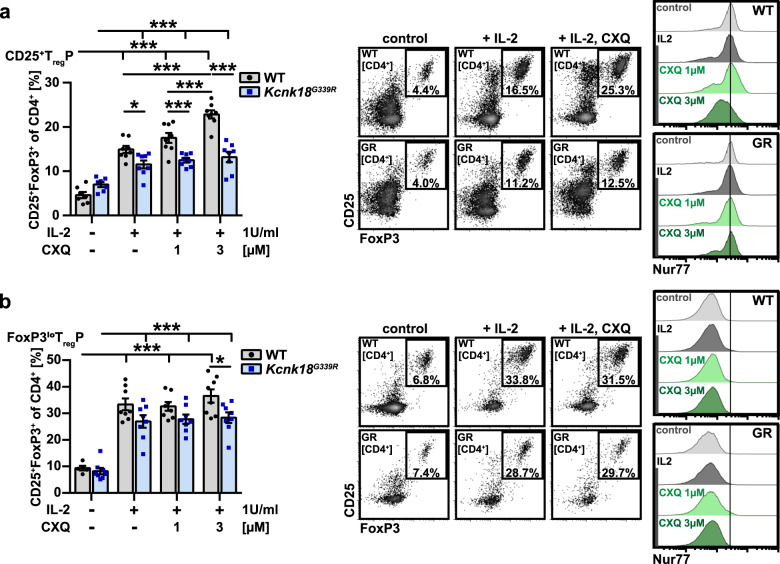
K_2P_18.1 specifically facilitates maturation of CD25^+^ TregP. **a**, **b** CD25^+^ TregP (**a**) and FoxP3^lo^ TregP (**b**) cells were isolated by cell sorting from WT and *Kcnk18*^*G339R*^*/Foxp3*^*RFP*^ thymus and stimulated for 72 h with 1 U/mL IL-2 and increasing concentrations of CXQ (*n* = 8). Left, Frequencies of converted tTreg; middle, representative FACS plots showing the Treg gate of the indicated conditions; right, histograms displaying Nur77 levels amongst converted tTreg cells. Midline represents median of Nur77 expression in WT CD25^+^ TregP cells. Data are represented as means ± SEM. *P < 0.05; **P < 0.01; ***P < 0.001.

Recently Owen et al.^39^ identified two distinct developmental programs for tTreg comprising CD25^+^ Treg progenitors (CD25^+^ TregP cells) and Foxp3^lo^ Treg progenitors (Foxp3^lo^ TregP cells). Therefore, we next questioned whether loss of K_2P_18.1 ion-current affects specific progenitor subsets. We found no differences in the proportions of CD25^+^ TregP cells between *Kcnk18*^*G339R*^ and WT mice and less mature TregP cells in *Kcnk18*^*G339R*^ mice, whereas FoxP3^lo^ TregP cells and more mature Treg progenitors already expressing FoxP3 were reduced (Fig. 7a; Supplementary information, Fig. S3c, d), which indicates that K_2P_18.1 ion-current is especially important at stages of FoxP3 induction in tTreg progenitors. In support of this hypothesis, kinase activity profiles showed substantial alterations in CD25^+^ TregP cells from *Kcnk18*^*G339R*^ mice (compared to WT mice, mainly comprising kinases involved in TCR, Ca^2+^ and NF-κB signaling), but not in FoxP3^lo^ TregP cells (Fig. 7b). CD25^+^ tTregP cells showed a stronger correlation between K_2P_18.1 and Nur77 expression (Fig. 7c) and higher expression levels of K_2P_18.1 compared to FoxP3^lo^ tTregP and mature tTreg cells (Figs. 7d, 6b). Nur77 expression is highly dependent on the intracellular Ca^2+^ signal.^40^ Consistent with an important role of K_2P_18.1 in Ca^2+^ signaling in tTreg progenitors, the correlation between Nur77 and K_2P_18.1 expression was nearly abrogated in *Kcnk18*^*G339R*^ mice (Fig. 7c). Moreover, K_2P_18.1 and Nur77 expression was reduced in *Kcnk18*^*G339R*^ mice compared to WT mice (Fig. 7d). The difference in Nur77 expression between *Kcnk18*^*G339R*^ and WT mice was pronounced in CD25^+^ tTregP cells, which supports that K_2P_18.1 is especially important at stages of FoxP3 induction in tTreg progenitors (Fig. 7d). In accordance, stimulation of TregP with IL-2 and increasing doses of cloxiquine (CXQ) (a K_2P_18.1 agonist, for further details see below) led to increased mature tTreg only in CD25^+^ TregP (Fig. 8a) and not in FoxP3^lo^ TregP cells (Fig. 8b). Further, this shows that K_2P_18.1 ion-current is important for the thymic selection process of tTreg as with increasing CXQ concentrations, tTreg populations with low Nur77 expression were increasingly selected. Thus, K_2P_18.1 channel function is especially important for tTreg developmental stages, where FoxP3 induction is required to proceed in maturation.

### Modulation of K_2P_18.1 function ameliorates autoimmune neuroinflammation in vivo

**Fig. 9:**
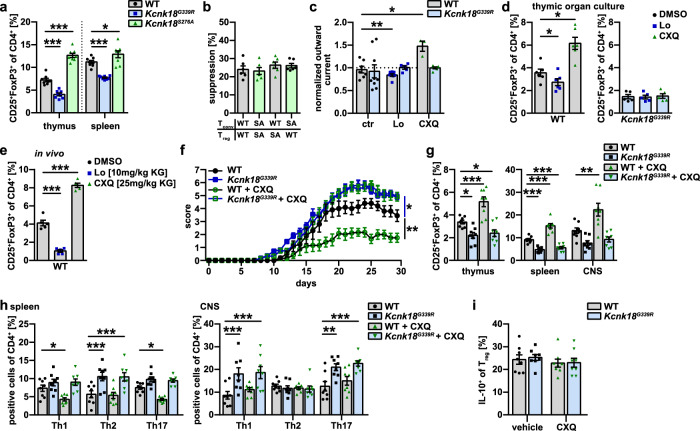
Modulation of K_2P_18.1 function allows to adjust Treg numbers and thereby to ameliorate autoimmune neuroinflammation. **a** Treg proportions in thymus and spleen from WT, *Kcnk18*^*G339R*^ and *Kcnk18*^*S276A*^ mice (*n* = 8 per group). **b** Suppression assay of proliferation of Tconv by Treg isolated from WT and *Kcnk18*^*S276A*^ mice (*n* = 6 per group). **c** Potassium outward currents in tTregs from WT-*Foxp3*^*RFP*^ and *Kcnk18*^*G339R*^*/Foxp3*^*RFP*^ mice with or without K_2P_18.1 inhibitor Lo (0.5 µM) or K_2P_18.1 activator CXQ (3 µM) treatment (*n* = 3–6). **d** E18 thymic organ cultures of WT and *Kcnk18*^*G339R*^ mice treated with Lo (0.5 µM) or CXQ (3 µM). Flow cytometry of tTreg frequencies in the thymus on day 7 of thymic organ culture (*n* = 6 per group). **e** Treg frequencies in the thymus of WT mice on day 7 after daily i.p. injection of Lo (10 mg/kg) or CXQ (25 mg/kg) (*n* = 5 per group). **f** Disease course of MOG_35–55_ EAE in WT and *Kcnk18*^*G339R*^ mice prophylactically (starting 7 days before immunization) treated with CXQ or vehicle (PBS) (*n* = 12 per group, two independent experiments). **g** Frequency of Treg in thymus, spleen and CNS of EAE mice (*n* = 8). **h** Frequency of Th1, Th2 and Th17 in spleen and CNS of EAE mice (*n* = 8 per group). **i** Intracellular cytokine staining for IL-10 in Tregs isolated from spleens of EAE mice. Data are represented as means ± SEM. *P < 0.05; **P < 0.01; ***P < 0.001.

Reduced numbers and/or impaired function of tTreg are pathophysiologic hallmarks of autoimmune disorders. We thus asked whether activation of K_2P_18.1 can increase tTreg numbers and whether this is meaningful in the context of autoimmunity in vivo. We generated a mutant mouse line carrying a single amino acid mutation, S276A, which disrupted the phosphorylation of the S276 residue (Supplementary information, Fig. S1c). In contrast to G339R, introducing the S276A point mutation into murine K_2P_18.1 results in increased (4.2-fold) basal K^+^ currents.^26^ Single positive CD4 thymocytes isolated from *Kcnk18*^*S276A*^ and *Kcnk18*^*G339R*^ mice partly expressed K_2P_18.1, whereas tTregs were all K_2P_18.1 positive and showed high K_2P_18.1 expression levels (Supplementary information, Fig. S6a, b). *Kcnk18*^*S276A*^ mice showed increased Treg numbers in thymus and spleen (Fig. 9a) and Treg function was not changed (Fig. 9b). These data further support an ion-current-mediated K_2P_18.1 effect on tTreg development and open up the possibility of a therapeutic application in the context of autoimmunity. To further investigate this possibility, we performed a detailed electrophysiological characterization of potential K_2P_18.1 modulators in K_2P_18.1-transfected HEK293T cells. CXQ and loratadine (Lo) were identified as most suitable activating and inhibiting agents, respectively (Supplementary information, Fig. 7a–k), and showed specific effects on K_2P_18.1-related potassium outward current in tTreg (Fig. 9c). In agreement with the results obtained in transgenic mice, K_2P_18.1 activation by CXQ facilitated Treg development, whereas inhibition by Lo reduced Treg numbers in thymic organ cultures (Fig. 9d). In contrast, thymic organ cultures of *Kcnk18*^*G339R*^ mice showed no changes of Treg numbers upon drug treatment (Fig. 9d), indicating a specific effect of K_2P_18.1. Treatment of WT mice with CXQ and Lo for 7 days mimicked the in vitro results and demonstrated that pharmacological modulation of tTreg development is possible under physiological conditions (Fig. 9e). Next, we assessed whether these findings are relevant in the context of autoimmunity in vivo using experimental autoimmune encephalitis (EAE) as a paradigmatic model. In EAE, loss of K_2P_18.1 ion-current in *Kcnk18*^*G339R*^ mice led to an aggravated disease course, whereas the prophylactic treatment with CXQ (starting 7 days before immunization to stimulate tTreg development) ameliorated disease severity (Fig. 9f). CXQ treatment had no impact on EAE disease course in *Kcnk18*^*G339R*^ mice, indicating a specific, K_2P_18.1 ion-current-dependent effect of CXQ (Fig. 9f). Consistent with a Treg-driven and K_2P_18.1-specific effect, CXQ treatment increased the proportions of Treg in thymus, spleen and CNS only in WT mice, and did not change those in *Kcnk18*^*G339R*^ mice (Fig. 9g). Compared to WT mice, *Kcnk18*^*G339R*^ mice showed decreased Treg proportions (Fig. 9g) and an increase of pathogenic Th1 and Th17 subsets in spleen and CNS (Fig. 9h). CXQ treatment had no obvious effect on Treg function as indicated by similar IL-10 production in the presence or absence of CXQ (Fig. 9i). Thus, Treg numbers can be dynamically adjusted by pharmacological modulation of K_2P_18.1 in mice, which can be exploited for the therapy of autoimmunity.

### K_2P_18.1 is involved in human tTreg development and autoimmunity

**Fig. 10:**
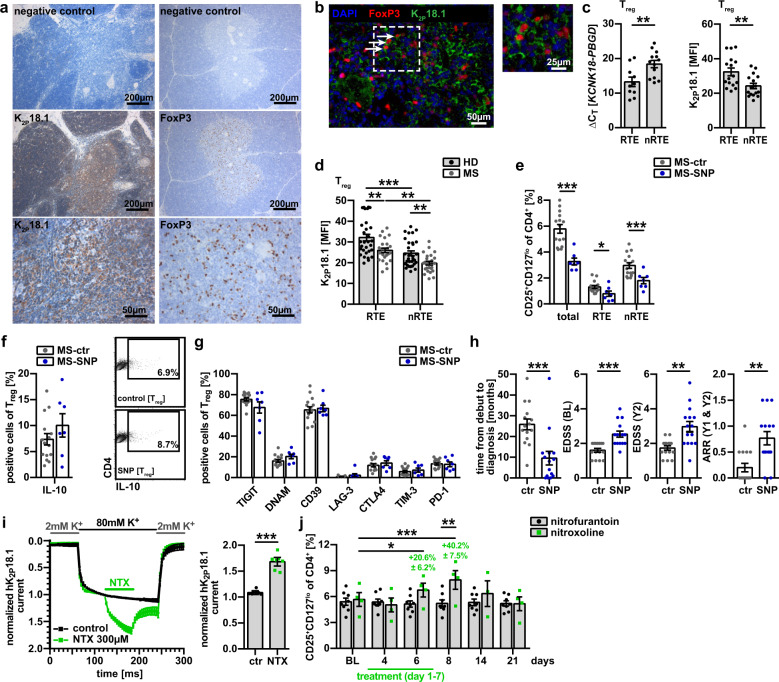
K_2P_18.1 in Treg development in human thymus. **a** Representative immunohistochemical staining of human thymus for K_2P_18.1 and FoxP3. Scale bars represent 200 µm or 50 µm, respectively (*n* = 3). **b** Representative immunofluorescence staining of human thymus for K_2P_18.1 (green), FoxP3 (red) and DAPI (blue). Inset shows marked area in higher magnification. Scale bars represent 50 µm or 25 µm, respectively (*n* = 3). **c** K_2P_18.1 mRNA and protein expression in sorted RTE Treg (CD4^+^CD8^−^CD31^+^CD25^high^CD127^lo^) and nRTE Treg (CD4^+^CD8^−^CD31^−^CD25^high^CD127^lo^) (*n* = 10 + 13). **d** Expression of K_2P_18.1 in RTE and nRTE Tregs from RRMS patients (MS, *n* = 25) and sex- and age-matched healthy controls (HD, *n* = 31). **e** Treg proportions in PBMCs from RRMS patients with (MS-SNP, *n* = 7) or without (MS-ctr, *n* = 15) the dominant-negative K_2P_18.1-C110R variant. **f** IL-10 production in Treg from RRMS patients with (MS-SNP, *n* = 7) or without (MS-ctr, *n* = 15) the dominant-negative K_2P_18.1-C110R variant. Left: bar graphs; right: representative dot plots. CD4^+^ T cells were used for gating on Treg (CD25^+^FoxP3^+^); IL-10 expression in Treg was assessed by plotting IL-10 against CD4. **g** Effector molecule expression in Treg from RRMS patients with (MS-SNP, *n* = 7) or without (MS-ctr, *n* = 15) the dominant-negative K_2P_18.1-C110R variant. **h** Clinical parameters of RRMS patients with (*n* = 15) or without (*n* = 15) the K_2P_18.1-C110R variant. EDSS, expanded disability status scale; ARR, annualized relapse rate; BL, baseline. **i** Normalized K_2P_18.1 current of oocytes transfected with hK_2P_2.1 in the presence of 300 µM nitroxoline (NTX, *n* = 7) or under control conditions (*n* = 6). Left, normalized hK_2P_18.1 current over time; Right, quantification of normalized current at the end of NTX application. Currents were normalized to the end of high-potassium buffer application. **j** Patients with UTI received nitroxoline (*n* = 4) or nitrofurantoin (*n* = 8) according to the summary of product characteristics for 7 days. Evaluation of Treg numbers by flow cytometry at the indicated time points. BL, baseline. Data are represented as means ± SEM. *P < 0.05; **P < 0.01; ***P < 0.001.

Next, we asked whether the findings in mice can be translated to humans. To test this, we investigated human thymus and blood samples. We found high expression of K_2P_18.1 and FoxP3 in the medulla of human thymus of healthy donors (HD) (Fig. 10a).^41,42^ In addition, most FoxP3^+^ cells in the thymic medulla co-expressed K_2P_18.1 (Fig. 10b). Moreover, human recent thymic emigrant (RTE) Treg (CD3^+^CD8^−^CD4^+^CD45RA^+^CD31^+^CD25^−^CD127^+^; Supplementary information, Fig. S8b) collected from peripheral blood mononuclear cells (PBMCs), expressed significantly more *KCNK18*/K_2P_18.1 than non-RTE (nRTE) Treg (CD3^+^CD8^−^CD4^+^CD45RA^−^CD31^−^CD25^hi^CD127^lo^) at both the mRNA and protein levels (Fig. 10c). Next, we investigated whether alterations in tTreg-related K_2P_18.1 function are relevant in the context of human autoimmunity. To do this, we compared the K_2P_18.1 protein expression on RTE and nRTE Tregs in relapsing-remitting multiple sclerosis (RRMS) patients versus HD. RRMS patients showed lower expression levels of K_2P_18.1 on both RTE and nRTE Tregs (Fig. 10d), suggesting that reduced K_2P_18.1 activity might be related to human autoimmunity. Interestingly, previous studies identified a single nucleotide polymorphism (SNP, rs140325655) coding for a dominant-negative missense K_2P_18.1 variant (K_2P_18.1-C110R) in which ion conductance is abolished.^43^ In a multicentre RRMS cohort, we identified 15 patients carrying the rs140325655 SNP. Cryopreserved PBMCs were available for seven of these patients. In accordance with our mouse data, the rs140325655 RRMS patients had significantly reduced numbers of Treg in the peripheral blood compared to RRMS controls (Fig. 10e). Functional parameters were not different between these two groups (Fig. 10f, g). However, rs140325655 RRMS patients showed more severe disability (EDSS) at baseline and 2-year follow up and significantly higher relapse rates (Fig. 10h). Cohort-specific (Münster versus Mainz) effects were not observed. A pharmacological activation of K_2P_18.1 might therefore have beneficial effects on human autoimmunity by increasing Treg numbers. For a first feasibility evaluation in humans, we searched for approved drugs with similar molecular structure to CXQ and identified nitroxoline, an antibiotic used to treat urinary tract infections (UTI) (Supplementary information, Fig. S9a).^44^ Nitroxoline significantly increased the ion-current through human K_2P_18.1 channels in an oocyte expression system (Fig. 10i). In UTI patients treated with nitroxoline or nitrofurantoin (another antibiotic used to treat UTI) for 7 days, we measured Treg numbers in the peripheral blood before, under and after therapy. Interestingly, only nitroxoline led to a rapid and reversible elevation of Treg numbers of up to 40.2% ± 7.5% compared to baseline (Fig. 10j). In vitro, nitroxoline had no impact on Treg proliferation unless toxic concentrations were reached (Supplementary information, Fig. S9b, c). In addition, Treg phenotype (Supplementary information, Fig. S9d) and function (Supplementary information, Fig. S9e) were similar to untreated conditions. Nitroxoline-treated subjects reported no adverse events except red discoloring of urine.

Overall, these results strongly support a similar role for K_2P_18.1 in the regulation of tTreg development and autoimmunity in humans, further underlining the clinical relevance of our findings and opening up the possibility to exploit this mechanism in therapeutic applications. Drug repurposing might allow for immediate clinical drug development.

## Discussion

tTreg development in thymus is a tightly regulated and highly efficient process. To trigger successful tTreg generation, TCR and consecutive intracellular Ca^2+^ signal strength need to reach a narrow corridor. However, the tuning mechanisms for those signals are largely unknown.

Here we found that genetic deletion of K_2P_18.1 induced a mild lymphoproliferative phenotype with reduced numbers of functional tTreg. The reduction of tTreg numbers was related to the loss of K_2P_18.1 ion-current function, which led to a defect in tTreg generation at developmental stages where FoxP3 expression is induced. K_2P_18.1 expression provided a specific selection advantage for tTreg progenitors and was coupled to TCR signal strength via NF-κB signaling. This advantage was provided by K_2P_18.1 via driving sustained Ca^2+^ influx inducing NF-κB- and NFAT-mediated FoxP3 expression. Pharmacological or genetic amplification of K_2P_18.1-mediated ion-current increased tTreg numbers and alleviated CNS autoimmunity. We observed a similar role for K_2P_18.1 in human tTreg development as well as in autoimmunity, and first in-human data showed the therapeutic potential of pharmacological K_2P_18.1 modulation.

The fact that K_2P_18.1-knockout mice and mice carrying a loss of ion-current function point mutation (G339R) presented the same phenotype of reduced tTreg, while the S276A point mutation, with augmented ion-current, led to increased tTreg numbers, strongly support our initial hypothesis that K^+^ conductance through K_2P_18.1 plays a crucial role in Treg development in the thymus.

Thymic selection of tTregs is limited by intraclonal competition for rare, tissue-specific self-antigens and Treg development seems to require higher TCR signaling strength than Tconv differentiation.^5^ Therefore, a prolonged Ca^2+^ signal mediated by K_2P_18.1 might facilitate TCR signal and Treg development. Similar to members of the tumor-necrosis factor receptor superfamily that couple TCR signal strength and tTreg development,^12^ Nur77 expression was positively correlated with K_2P_18.1 expression in tTreg progenitors. Furthermore, higher K_2P_18.1 expression resulted in a selective advantage to undergo maturation. We identified that the NF-κB pathway directly links TCR signaling and K_2P_18.1 expression.

Consistent with distinct developmental programs for different tTreg progenitor subsets,^39^ we found that the correlation between Nur77 and K_2P_18.1 was much stronger for CD25^+^ TregP cells than for FoxP3^lo^ TregP cells and loss of K_2P_18.1 ion-current led to a pronounced reduction of Nur77 expression levels in CD25^+^ TregP cells. Furthermore, the kinome of CD25^+^ TregP cells was significantly altered in *Kcnk18*^*G339R*^ mice, whereas only slight changes were observed for that of FoxP3^lo^ TregP cells. Pharmacological activation of K_2P_18.1 increased mature tTreg only in CD25^+^ TregP cells and not in FoxP3^lo^ TregP cells. Cumulatively, these data indicate a specific selection advantage by high K_2P_18.1 expression in CD25^+^ TregP cells. However, as we observed reduced proportions at all tTreg developmental stages that require induction and expression of FoxP3 including FoxP3^lo^ TregP cells, this role of K_2P_18.1 seems to involve further tTreg progenitors. For FoxP3^lo^ TregP cells, those have not been identified so far.^39^ Thus, K_2P_18.1 expression represents an important factor defining the Treg developmental niche decoding TCR signaling strength and facilitating FoxP3 expression.

Previous studies revealed that variations in TCR signaling strength are encoded as specific patterns of [Ca^2+^]_i_ dynamics and then decoded into differential lymphocyte fates and functions.^34,35,45^ Ca^2+^-regulated transcription factors including NF-κB and NFAT decode those signals in lymphocytes and are essential for induction and stabilization of FoxP3 expression in tTreg.^11,13,46,47^ Loss of K_2P_18.1 ion-current function reduced nuclear translocation of both NF-κB and NFAT. External TCR stimulation was able to compensate for reduced NF-κB signaling. Thus, K_2P_18.1 ion-current function is even more important for NFAT-related cellular signals. This might be related to differential tuning of those transcription factors by [Ca^2+^]_i_. NF-κB is activated by one or few transient cytoplasmic Ca^2+^ spikes, whereas NFAT activation requires a sustained increase in [Ca^2+^]_i._^32,33^ To achieve such high Ca^2+^ signals for NFAT activation in tTreg, we postulate a positive feedback loop involving TCR, NF-κB and K_2P_18.1 in which TCR-induced transient Ca^2+^ spikes activate NF-κB, which in turn facilitates K_2P_18.1 expression. Upregulation of this K^+^ conductance hyperpolarizes tTreg and thus enhances the driving force for sustained Ca^2+^ entry. Following tTreg maturation, loss of K_2P_18.1 might have no significant impact on Treg function since Treg does not require NFAT to suppress effector immune cells.^48–50^

Although bulk RNA-sequencing of tTreg suggested effects on cell cycle and apoptosis by loss of K_2P_18.1 ion-current function, subsequent functional experiments did not confirm these findings, which does not exclude discrete alterations that we were not able to detect. In addition, proliferation of tTreg was not obviously affected by loss of K_2P_18.1 function; however, further experiments might be required to exclude this. Furthermore, despite the observed lymphoproliferative phenotype, markers indicative of T cell activation and proliferation showed only slight alterations in Tconv from *Kcnk18*^−*/*−^ mice, while not reaching statistical significance. These data are consistent with the observed lymphoproliferative phenotype, but the used markers seem not to be sensitive enough for the clear detection of effects on immune cell activation.

Treg cell research has impressively progressed over the past two decades. However, most knowledge is based on observations in animal models and studies in humans are sparse.^42^ To the best of our knowledge, this is the first report of a potential role for K_2P_18.1 in human tTreg development. We found that FoxP3^+^ thymocytes co-expressing K_2P_18.1 were predominantly in the medullary region of the human thymus, which is of particular relevance to Treg development.^41,42^ Furthermore, we observed higher K_2P_18.1 expression levels in RTE Treg compared to nRTE Treg. RTEs have been shown to be preferential precursors of Tregs in the periphery.^51^ In the context of human autoimmunity, such as in MS, K_2P_18.1 expression was reduced on both Treg subtypes. Whether this is a pathogenic feature and/or the consequence of MS remains elusive, however, these findings further support a central role of ion channels in MS.^52^ In strong support of a critical role of K_2P_18.1 in Treg-related immune tolerance and MS, MS patients carrying the missense K_2P_18.1 variant rs140325655, with abolished ion conductance, presented reduced Treg numbers in peripheral blood and poor clinical outcomes. Although this serves as a proof of principle, it remains elusive whether rs140325655 represents a relevant genetic risk variant in other MS cohorts.

We here showed that pharmacological modulators (activator: CXQ; blocker: Lo) are able to modulate K_2P_18.1 function, thereby adjusting Treg numbers with clear impact on autoimmunity in vivo. We did not observe alterations of tTreg numbers in *Kcnk18*^*G339R*^ mice upon treatment with CXQ and Lo either in vitro or in vivo, arguing for specific effects of K_2P_18.1. However, as Lo is mainly known as histamine 1 receptor antagonist, further drug development is needed to reduce off-target effects. A pharmacological method capable of dynamically modulating Treg numbers might avoid the shortcomings of cell based-approaches such as high costs, long time required, low cell yield and extensive precautions.^53–55^ Moreover, our results from transgenic mice (e.g., CD4^+^ cell-specific *Kcnk18*^−*/*−^ mice, major immune cell subsets in thymus and spleen, TCR repertoire of Tconv) argue for specific effects of K_2P_18.1 modulation on the tTreg compartment, which is in contrast to previously used Treg-inducing drugs such as rapamycin that broadly affects the immune system.^56^

Based on CXQ we searched for approved drugs with similar molecular structures and identified nitroxoline, an antibiotic used to treat UTI. Nitroxoline activated human K_2P_18.1 channel function in expression models and consistent with to our mouse data, led to a rapid and reversible elevation of Treg numbers with unaltered function in the peripheral blood of patients with UTI. Of note, nitrofurantoin, another antibiotic used to treat UTI, showed no effect on Treg numbers, arguing for a rather specific effect of nitroxoline and no major influence of the infection itself. The therapeutic implications of our findings might be far-reaching since a rapid and dynamic regulation of Treg numbers will be useful in a large variety of human disorders including autoimmunity, malignoma, sepsis, organ transplant or COVID-19 infection characterized by a viral response and a host (hyper)inflammatory disease phase.^57^ However, the low sample size does not allow for definitive conclusions and we cannot formally rule out off-target effects of nitroxoline on other K_2P_18.1-expressing cells affecting Treg numbers.

In conclusion, our data support an important role for K_2P_18.1 in tTreg development by a multistep mechanism: (1) TCR activation leads to upregulation of K_2P_18.1 expression via NF-κB; (2) high K_2P_18.1 expression allows for a selection advantage that enables Treg progenitors to undergo maturation; (3) K_2P_18.1-related intracellular Ca^2+^ signals facilitate NF-κB and NFAT signaling, forming a positive feedback loop that promotes FoxP3 induction and stabilization (Supplementary information, Fig. S1e). Thus, K_2P_18.1-related Ca^2+^ dynamics drive tTreg fate and define the tTreg developmental niche. Pharmacological modulation of K_2P_18.1-mediated Ca^2+^ signaling allows for dynamic regulation of tTreg numbers, suggesting that therapeutic administration of K_2P_18.1-modulating agents could treat a wide range of pathological conditions. First in human experiments with a K_2P_18.1 activator support further clinical development.

## Materials and methods

Software for data acquisition and analysis and a detailed list of antibodies used in this study can be found in Supplementary information, Data S1.

### Mice

All animal studies were approved by institutional care committee and state committees for animal welfare (84-02.04.2016_A307, A17.019). Animal experiments were conducted in accordance with the European Union normative for care and use of experimental animals and the German Animal Protection Law. Mice were raised in an in-house animal facility or purchased from Charles River and Jackson Laboratories and kept in individually ventilated cages under specific pathogen-free conditions and fed ad libitum. All mice were on a C57BL/6 background. Transgenic mouse lines were bred to generate homozygous mice. Mice were used at the age of 8–12 weeks for all experiments, if not stated otherwise.

#### Active MOG-EAE

Induction of EAE was performed in 8–12-week-old female mice as previously described.^20,58^ Briefly, MOG_35–55_ peptide was dissolved in phosphate-buffered saline (PBS) (2 mg/mL) and homogenized with complete Freund’s Adjuvant (CFA, 2 mg/mL) at 1:1 ratio and stored for 30 min at 4 °C. 100 µL MOG emulsion was injected into each flank of anesthetized mice (isoflurane). Injection of pertussis toxin (PTX, 1 µg/µL) was performed on day 0 and day 2 after intraperitoneal (i.p.) immunization with MOG (200 µL per dose). Health status (weight, disease score, general appearance and performance) of mice was monitored on a daily basis. Treatment of WT and *Kcnk18*^*G339R*^ mice with CXQ was performed by i.p. injection of 25 mg/kg body weight CXQ (in 100 µL PBS, DMSO) or vehicle alone daily, starting 1 week before immunization.

#### Generation of *Kcnk18*^−*/*−^ mice

CRISPR-Cas9 system was used to generate *Kcnk18*^−*/*−^ mice.^59^ The gRNA target was selected in the protein-coding part of exon 3 of the *Kcnk18* gene. This gRNA was cloned into the plasmid gRNA_Cloning Vector (Addgene plasmid# 41824) digested with the *Afl*II restriction endonuclease using Gibson assembly with help of oligonucleotide pairs: KCNK18_Insert_F2/KCNK18_Insert_R2, resulting in plasmid pgRNA_KCNK18cas2. CV19 ES cells (passage 13 (129SV × C57BL/6J)) were expanded and the linearized targeting vector DNA pKCNK18_targ (100 µg) was electroporated together with 70 µg of each plasmid DNA: pgRNA_KCNKcas2 and hCAS9 (Addgene plasmid# 41815) at 25 µF and 400 V in 0.8-mm electroporation cuvettes (Gene Pulser; Bio-Rad). After electroporation, cells were cultivated for 10 min at room temperature (RT) and plated onto ten 100-mm diameter culture dishes containing a γ-irradiated monolayer of mouse primary G418-resistant fibroblast feeder cells. After 32 h, 350 µg/mL G418 and 0.2 µM FIAU (2′-deoxy-2′-fluoro-β-D-arabinofuranosyl-5-iodouracil) were added to the culture medium. The medium was replaced every day, and colonies were picked and analyzed 8 days after plating. Positively-targeted ES cell clones were analyzed using Southern blot. Correctly-targeted ES cells from two independent clones (E1 and D2) were injected into 3.5-day B6D2F1 blastocysts. Routinely, 12–14 ES cells were injected into one blastocoel. After injection, the blastocoel was transferred into the uteri of 2.5-day pseudopregnant CD-1 foster mice. The mice carried pups to term. Positively-targeted F0 and F1 animals were identified by qPCR and sequencing analysis of genomic DNA isolated from tail biopsies. The subjects were male and female *Kcnk18*-deficient mice (*Kcnk18*^*+/−*^) and the mouse line was established by breeding male with female C57Bl/6J mice to produce heterozygous mice. Subsequently, heterozygous mice were interbred to achieve *Kcnk18*^−*/*−^ homozygosity. In experiments WT littermates were used as controls.

#### Generation of *Kcnk18*^*G339R*^ mice

The G339R mutation, located in the selectivity filter of K_2P_18.1, demonstrates a loss of ion channel function. The G339R point mutation was obtained by random mutagenesis in C3HeB/FeJ mice as previously described.^30^ The C3HeB/FeJ-*Kcnk18*^*G339R*^ mice were contributed by AG Molecular Electrophysiology (Institute of Physiology Würzburg, Germany), backcrossed to C57Bl/6 for ten generations and kept as homozygous lines. In experiments WT littermates were used as controls.

#### Generation of *Kcnk18*^*S276A*^ mice

The S276A mutation in murine K_2P_18.1 leads to highly increased basal K_2P_18.1 K^+^ currents (4.2-fold increase) and was proposed to be the main amino acid residue responsible for K_2P_18.1 Ca^2+^ sensitivity.^26^ The *Kcnk18*^*S276A*^ mouse line was generated by direct oocyte microinjections using the CRISPR-Cas9 components together with the donor DNA oligo (Kcnk18_mutREV) followed by subsequent chirurgical embryo transfer. For the preparation of CRISPR-Cas9 microinjection solution, commercially synthesized Kcnk18_crRNA4 (CAGATTGCTGAGAATAGAAC), together with the tracrRNA and dCas9 protein were mixed as follows: 200 pmol of crRNA were mixed with 200 pmol of tracrRNA in 10 mM potassium acetate and 3 mM HEPES buffer (pH 7.5) and incubated at 95 °C for 2 min, followed by cooling to RT. The annealed crRNA/tracrRNA complexes were mixed with Cas9 mRNA, Cas9 protein, and Kcnk18_mutREV template DNA oligo. Microinjections were performed in B6D2F1 (hybrid between C57BL/6J and DBA strains) fertilized one-cell oocytes. Oocytes were removed from oviducts of super-ovulated B6D2F1 female mice in M2 media supplemented with hyaluronidase (400 µg/mL). Cytoplasmic microinjections were performed in M2 media using the Transjector 5246 (Eppendorf), and Narishige NT-88NE micromanipulators attached to a Nikon Diaphot 300 inverted microscope. Oocytes that survived microinjections were transferred to oviducts of pseudopregnant CD1 foster mice and carried to term. Positively-targeted F0 and F1 animals were identified by qPCR and sequencing analysis of genomic DNA isolated from tail biopsies. The subjects were males and females of *Kcnk18*^*S276A*^ mice. The mouse line was established by breeding male with female C57BL/6J mice to produce heterozygous mice. Subsequently, heterozygous mice were interbred to achieve *Kcnk18*^*S276A*^ homozygosity. In experiments WT littermates were used as controls.

#### Generation of *Kcnk18*^*fl/f*^ mice

To induce a cell type-specific deletion of K_2P_18.1, we generated a *Kcnk18*-floxed (*Kcnk18*^*fl/fl*^) mouse line that was then crossbred to a CD4cre mouse line to delete K_2P_18.1 specifically in CD4^+^ cells. The *Kcnk18* gene targeting construct for insertion of the loxP sites (pKCNK18_targ.) was designed as follows: the 3.5 kb left flanking region containing intron 2 genomic sequences was PCR amplified from mouse genomic DNA using oligonucleotides KCN_FLAdn (GTCTCAAGCGTCTCTTGGAGCGGCCGCAAGGCCTCAAATCCCTGATGTAGT) and KCN_FLArn (GCTCTAGACGTCTCTGAGAGGATCCGACAGGCAGATAAGAAAGAAGAAAGG), and subcloned. The 1.8 kb right flanking region containing non-protein-coding part of exon 3 genomic sequences was PCR amplified using oligonucleotides KCN_FLBdn (GTCTCAAGCGTCTCTTGGACGCGTCTTCCCACACCTTGGTTTTATACAG) and KCN_FLBrn (GCTCTAGACGTCTCTGAGAGTCGACATAGATGATAGATAACCAAGAAAGAAAG) and consequently subcloned. The 1.0 kb protein-coding part of the exon 3 together with intronic sequences was PCR amplified using oligonucleotides KCN_ex3dn (GTCTCAAGCGTCTCTTGGAATTCAGGATGAGTAATTTTTGCTGTGTAA) and KCN_ex3rn (GCTCTAGACGTCTCTGAGACGCGTATAACTTCGTATAATGTATGCTATACGAAGTTATGGTACCTCACATCTGTAGGTCACGGAAA), and subcloned. The protein-coding part of the exon 3 flanking *Lox*P site together with the *Kpn*I site was introduced by PCR cloning with help of the oligonucleotide KCN_ex3rn. All individual clones were verified by sequencing and assembled into the final targeting construct. The pBluescript-based plasmid backbone together with the negative selection marker (thymidine kinase cassette and diphtheria toxin gene), were added to the left flanking region. The positive selection marker (neomycin cassette flanked by two FRT sites and one *Lox*P site), was cloned as *Eco*RI–*Bam*HI DNA fragment between left flanking region and 1.0 kb protein-coding part of exon 3 DNA fragment. The gRNA target was selected from the protein-coding part of exon 3 of the *Kcnk18* gene. This gRNA was cloned into plasmid gRNA_Cloning Vector (gift from George Church (Addgene plasmid# 41824)) digested with the *Afl*II restriction endonuclease, using Gibson assembly with help of oligonucleotide pair: KCNK_Insert_F2 (TTTCTTGGCTTTATATATCTTGTGGAAAGGACGAAACACCGAGATTGCTGAGAATAGAAC)/KCNK_Insert_R2 (GACTAGCCTTATTTTAACTTGCTATTTCTAGCTCTAAAACGTTCTATTCTCAGCAATCTC), resulting in plasmid pgRNA_KCNKcas2. CV19 ES cells (passage 13 (129Sv × C57BL/6J)) were expanded in HEPES-buffered Dulbecco’s modified Eagle’s medium (DMEM) supplemented with 15% fetal bovine serum (FBS), non-essential amino acids, L-glutamine, β-mercaptoethanol, 1000 U/mL recombinant leukemia inhibitory factor (MERCK Millipore), and antibiotics (100 U/mL penicillin and 100 U/mL streptomycin). For electroporation, 2 × 10^7^ cells were resuspended in 0.8 mL Capecchi buffer (20 mM HEPES, pH 7.4, 173 mM NaCl, 5 mM KCl, 0.7 mM Na_2_HPO_4_, 6 mM dextrose, 0.1 mM β-mercaptoethanol). The *Not*I-linearized targeting vector DNA pKCNK18_targ. (100 µg) was electroporated together with 70 µg of each plasmid DNA, pgRNA_KCNKcas2 and hCAS9 (cas9 coding plasmid was a gift from George Church (Addgene plasmid# 41815)) at 25 µF and 400 V in 0.8 mm electroporation cuvettes (Gene Pulser; Bio-Rad). After electroporation, cells were cultivated for 10 min at RT and plated onto ten 100-mm diameter culture dishes containing a γ-irradiated monolayer of mouse primary G418-resistant fibroblast feeder cells. Thirty-two hours later, 350 µg/mL G418 (Invitrogen) and 0.2 µM FIAU (Moravek Biochemicals and Radiochemicals, USA) were added to the culture medium. The medium was replaced every day, and colonies were picked and analyzed 8 days after plating. Correctly-targeted ES cells from two independent clones (E1 and D2) were injected into 3.5-day B6D2F1 blastocysts. Routinely, we are injecting 12–14 ES cells into one blastocoele. After injection, blastocysts were kept in KSOM medium and subsequently transferred into the uteri of 2.5-day pseudopregnant CD-1 foster mice. The mice carried pups to term. Chimeras were identified by their agouti coat color contribution. For the germline transmission, high percentage male chimaeras were crossed to the C57BL/6J female mice. Heterozygous agouti offspring (*Kcnk18*^*fl/−*^) were confirmed by Southern blot analysis and further tested by PCR for the presence of the targeted allele. The FLPe-mediated neo cassette excision was performed in vivo by cross-breeding mice with the FLPe transgene, resulting in heterozygous *Kcnk18*-floxed mice (*Kcnk18*^*fl/+*^). Mice were kept in specific pathogen-free animal facilities. All mouse procedures were performed in compliance with the guidelines for the welfare of experimental animals issued by the Federal Government of Germany. The mouse line was established by breeding male with female C57Bl/6J mice to produce heterozygous mice. Subsequently, heterozygous mice were interbred to achieve *Kcnk18*^*fl/fl*^ homozygosity.

### Primary cell isolation

#### Leukocyte isolation

Organs (mouse spleen, thymus and lymph node) were homogenized by a 40 µm cell strainer and washed with 10 mL washing medium (DMEM, 1% FCS, 1% penicillin/streptomycin). Erythrocytes in the splenocyte suspension were lysed with ACK buffer (150 mM NH_4_Cl, 10 mM KHCO_3_, 0.1 mM EDTA, pH 7.3) for 30 s, stopped by addition of washing medium. Single cell suspensions were washed once again and resuspended in the desired buffer for subsequent applications.

#### Isolation of CD4^+^ T cells and Treg

To isolate CD4^+^ T cells and Treg from spleen or lymph node, CD4^+^ T Cell Isolation Kit (MACS, Miltenyi Biotec) or CD4^+^CD25^+^ Regulatory T Cell Isolation Kit (MACS, Miltenyi Biotec) were used according to the manufacturer’s protocol. In brief, single cell suspensions were incubated with the CD4^+^ T cell biotin–antibody cocktail for 5 min (4 °C), followed by the incubation with anti-biotin MicroBeads for 10 min (4 °C). Labeled cells were separated from the unlabeled CD4^+^ T cell population by magnetic field separation. For further Treg isolation, cells were labeled by a CD25-PE antibody and incubated with anti-PE MicroBeads (each step 15 min, 4 °C). Alternatively, CD4^+^ T cells and Treg (CD3^+^CD4^+^CD8^–^CD25^+^FoxP3^+^) were isolated by FACS sorting of *Foxp3*^*RFP*^ mice, counterstained for CD3, CD4, CD25 and CD8a.

#### Isolation of thymic APC and mTEC

Thymus of WT and *Kcnk18*^*G339R*^ mice was homogenized by enzymatic digestion. Therefore, the thymus was dissected into small pieces by cutting with scissors in 5 mL RPMI containing 2% FCS. 0.5 mg/mL collagenase D and 20 µg/mL DNase I were added to the tissue and incubated for 45 min at 37 °C on an orbital shaker. Digestion was stopped by 5 mL of 10 mM EDTA, followed by incubation for 5 min at RT. Thymus homogenate was then rinsed through a 70 µm cell strainer and washed with 20 mL RPMI + 2% FCS. To isolate CD11c^+^ cells, CD11c MicroBeads (MACS, Miltenyi Biotec) were used in a MACS separation according to the manufacturer’s protocol. After isolation, cells were stained for flow cytometry with antibodies directed to CD8, CD11b, CD11c, CD45R/B220, CD86, MHC-II, SIRPα. For CD45^–^ mTEC isolation, we used the CD11c^–^ flow-through from CD11c MACS isolation in a negative selection using CD45 MicroBeads (MACS, Miltenyi Biotec) according to the manufacturer’s protocol. After MACS isolation, CD45^–^ mTEC was used for flow cytometry analysis (CD45, CD80, BP-1, EpCAM, MHC-II).

### Proliferation assay

Splenocytes were isolated as described before. Then cells were labeled with Vybrant^TM^ CFDA SE Cell Tracer (12.5 µM) in 2 mL PBS + 2% FCS for 10 min at 37 °C, followed by addition of 10 mL cold washing buffer and incubation on ice for 10 min. Afterwards splenocytes were washed and seeded into 96-well plates (U-bottom) coated with different concentrations of anti-CD3. Soluble anti-CD28 was added to the splenocyte complete medium (DMEM, 10 mM HEPES, 25 µg/mL gentamycin, 5 µM β-mercaptoethanol, 1% non-essential amino acids, 5% FCS) as indicated in the respective experiments. Cells were cultured for 3 days (37 °C, 5% CO_2_) prior to FACS analysis.

The proliferation of human CD4^+^ T cells was assessed by labeling the cells with CFSE as described before.^58^ Cells were cultured in the presence of plate-bound anti-CD3 (1 µg/mL), soluble anti-CD28 (2 µg/mL) and nitroxoline at different concentrations for up to 7 days (37 °C, 5% CO_2_) prior to FACS analysis.

### Suppression assay

Splenocytes were isolated as described before. CD4^+^CD25^–^ Tconv and CD4^+^CD25^+^ Treg were isolated as described above using the CD4^+^CD25^+^ Regulatory T cell Isolation Kit (MACS, Miltenyi Biotec). Tconv were stained with CFSE to track cell proliferation and co-cultured with Treg (4:1 ratio, stimulation with 0.5 µg/mL plate-bound anti-CD3, 1 µg/mL soluble anti-CD28). Cells were cultured for 3 days (37 °C, 5% CO_2_) prior to FACS analysis. For antigen-specific suppression assays, Treg from the respective mouse line and Tconv from 2D2 mice were isolated and labeled with CFSE as described before. For isolation of APCs from WT mice, the spleen was homogenized by enzymatic digestion. Therefore, 0.1 mg/mL collagenase D was injected into the spleen and incubated for 15 min at 37 °C. Spleen was homogenized by a 70 µm cell strainer. To isolate CD11c^+^ APCs, CD11c MicroBeads (MACS, Miltenyi Biotec) were used in a MACS separation according to the manufacturer’s protocol. APCs were incubated with 20 µg/mL MOG_35–55_ for 10 min at 37 °C and washed with PBS before setting up the culture. A co-culture of 5 × 10^4^ APCs and in total 2 × 10^5^ Tconv and Treg (ratios of 1:1, 2:1 and 4:1) was set up for 3 days. Flow cytometry analysis of CFSE-labeled Tconv was used to determine the suppressive capacity of Treg.

### In vitro Treg induction

Splenocytes were used for isolation of CD4^+^CD62L^+^ naïve T cells by the CD4^+^CD62L^+^ T Cell MACS Isolation Kit (MACS, Miltenyi Biotec). Then CD4^+^CD62L^+^ T cells were seeded into 96-well plates (U-bottom) in the presence of 2 µg/mL plate-bound anti-CD3 and 1 µg/mL soluble anti-CD28, 10 µg/mL anti-IL-4, 10 µg/mL anti-IFN-γ and 5 ng/mL TGFβ in mouse T cell medium (IMDM, 10% FCS, 1% L-glutamine, 5 µM β-mercaptoethanol) for 3 days (37 °C, 5% CO_2_) prior to FACS analysis.

### In vitro Th1, Th2 and Th17 induction

Splenocytes were used for isolation of CD4^+^CD25^*–*^CD44^−^ naïve T cells using the naïve CD4^+^ T Cell MACS Isolation Kit (MACS, Miltenyi Biotec). Then CD4^+^CD25^*–*^CD44^*–*^ T cells were seeded into 96-well plates (U-bottom) in the presence of 2 µg/mL plate-bound anti-CD3 and 4 µg/mL soluble anti-CD28. For Th1 induction, 200 U/mL IL-12, 50 U/mL IL-2 and 10 µg/mL anti-IL-4; for Th2 induction, 200 U/mL IL-4, 50 U/mL IL-2 and 10 µg/mL anti-IFNγ; for Th17 induction, 10,000 U/mL IL-6, 40 U/mL IL-23, 8400 U/mL IL-1b, 10 U/mL human-TGFβ1, 10 µg/mL anti-IL-4, 10 µg/mL anti-IFNγ and 10 µg/mL anti-IL-2 were added to the respective culture in mouse T cell medium (IMDM, 10% FCS, 1% L-glutamine, 5 µM β-mercaptoethanol) for 3 days (37 °C, 5% CO_2_) prior to FACS analysis.

### Thymic Treg induction from progenitor cells

Thymic Treg progenitor cells (CD3^+^CD4^+^CD8^−^CD25^+^FoxP3^−^ TregP and CD3^+^CD4^+^CD8^−^CD25^–^FoxP3^lo^ TregP) were isolated by FACS sorting. Cells were then cultured in mouse T cell medium with or without low-dose IL-2 (1 U/mL) in the presence of different concentrations of CXQ (1 µM, 3 µM) for 3 days (37 °C, 5% CO_2_). Differentiated cells were stained for Treg cell markers (CD3^+^CD4^+^CD25^+^FoxP3^+^) for quantification of mature tTreg induction and for Nur77 to assess TCR activation by flow cytometry.

### Immunophenotyping

Phenotyping of different immune cell subsets was performed using flow cytometry. Therefore, immune cell subsets from spleen and thymus were characterized by staining for CD3, CD4, CD8, CD11b, CD11c, CD19, CD45R/B220 and NK1.1 and CD44, CD62L and CD69 to assess their activation status. For characterization of the T-helper cell subsets Th1, Th2 and Th17, cells were intracellularly stained for their respective signature cytokines IFNγ, IL-4 and IL-17A. Further characterization of Tregs was performed by staining for CD3, CD4, CD8, CD25, FoxP3, Helios, IL-10, CD39, CD73, GITR (CD357), ICOS (CD278), TIGIT, CTLA-4 (CD152) and LAG3 (CD223). Thymic T cell developmental stages were assessed by staining for CD3, CD4, CD5, CD8, CD44, CD45R/B220, CD69 and TCRβ: DN (CD3^+^CD4^−^CD8^−^ double-negative), DP (CD3^+^CD4^+^CD8^+^ double-positive), SP4 (CD3^+^CD4^+^CD8^−^ single positive) and SP8 (CD3^+^CD4^−^CD8^+^ single positive) cell subsets. Development stages of SP4 cells towards tTreg were evaluated in *Foxp3*^*RFP*^ mice by staining for CD3, CD4, CD8, CD25, CD69, CCR7 and CCR9. Thymic APCs were characterized using antibodies directed to CD11b, CD11c, CD45R/B220, CD86, MHC-II and SIRPα; mTEC by CD45, CD80 BP-1, EpCAM and MHC-II.

### Cell cycle analysis

Thymic CD4^+^ T cells were isolated with CD4^+^ T Cell Isolation Kit (MACS, Miltenyi Biotec) as described above. Cells were analyzed directly after isolation and after 24 h and 48 h of stimulation (plate-bound anti-CD3 (2 µg/mL), anti-CD28 (4 µg/mL) in solution). Then cells were stained for CD4, CD8 and FoxP3 to gate for Treg cells as well as for PCNA and PI to analyze the cell cycle with flow cytometry. Cells in G0-phase were PCNA^−^/PI^−^, in G1-phase PCNA^lo^/PI^lo^, in S-phase PCNA^+^/PI^int^ and in G2-phase PCNA^lo/−^/PI^+^.

### Apoptosis analysis

CD4^+^ T cells from spleen and thymus were isolated with CD4^+^ T Cell Isolation Kit (MACS, Miltenyi Biotec). Cells were stained with CD3, CD4, CD8, CD25, FoxP3, fixable viability dye eFluor780 (FVD780) and Fam-FLICA-DEVD (FAM-FLICA Caspase 3 & 7 Assay Kit, LKT Labs) to analyze proportion of apoptotic cells. Caspase3/7^+^FVD^−^ cells were defined as early apoptotic and Caspase3/7^+^FVD^+^ cells as late apoptotic.

### Transcription factor staining

CD4^+^ T cells from thymus were isolated with CD4^+^ Isolation Kit (MACS, Miltenyi Biotec). Cells were stimulated for 3 h with 2 µg/mL anti-CD3 (plate-bound) and 4 µg/mL soluble anti-CD28 in mouse T cell medium. After harvest, cells were stained for Treg marker and transcription factors (NFAT, NF-κB, pSTAT5, pAkt). Mean fluorescence intensity (MFI), measured by flow cytometry, was used for pSTAT5 and pAkt as a measure for activation of STAT5 and Akt. Translocation of NFAT and NF-κB was assessed by ImageStream (AMNIS INSPIRE acquisition software, Luminex) and translocation score was calculated using nuclear translocation wizard in AMNIS IDEA software (Luminex).

### Analysis of K_2P_18.1 and Nur77 expression

CD4^+^ T cells were stained for CD3, CD4, CD8, CD25, FoxP3, K_2P_18.1 (anti-TRESK (E-2) monoclonal antibody (Santa Cruz, sc-514525), followed by secondary antibody staining) and Nur77, and analyzed by flow cytometry. For assessment of K_2P_18.1 expression upon TCR stimulation, CD4^+^ T cells were stimulated for 24 h with and without plate-bound anti-CD3 (0, 0.1, 0.5, 1 and 2 µg/mL) in mouse T cell media in the presence of soluble anti-CD28 (2 µg/mL) in all conditions. Thereafter cells were stained for CD3, CD4, CD8, CD25, FoxP3 and K_2P_18.1 and analyzed by flow cytometry.

### Blocking NF-kB signaling

CD4^+^CD25^+^ cells were isolated from WT thymus and stimulated with 2 µg/mL plate-bound anti-CD3 and 4 µg/mL soluble anti-CD28 for 3 h at 37 °C, 5% CO_2_. 20 µM CDN (Sigma-Aldrich), 5 µM PTN (Sigma-Aldrich) or DMSO was added to the cell culture during stimulation and in the unstimulated conditions. Cells were collected after 3 h and prepared for RNA isolation.

### Calcium imaging

CD4^+^ T cells were plated on 18 mm coverslips pre-coated with Poly-D-Lysine (PDL) at a density of 3 × 10^6^ cells per coverslip. Cells were centrifuged at 300 × *g* for 2 min and subsequently processed for imaging experiments without resting. This allowed attachment of the cells to the coverslip throughout the experiment. No movement of the cells was observed after change of solutions within the recording chamber. T cells were loaded for 30 min with fura-2 AM and 0.005% Pluronic (Sigma-Aldrich) in a HEPES-buffered solution (artificial CSF, containing 125 mM NaCl, 2.5 mM KCl, 1.25 mM NaH_2_PO_4_, 10 mM glucose, 2 mM MgSO_4_, 2 mM CaCl_2_ and 30 mM HEPES, pH 7.35, osmotic concentration of 305 mOsmol/kg). Prior to experiments, PDL was removed and coverslips were washed with the HEPES-buffered solution, which then served as the extracellular solution throughout the experiment. Prior to the Ca^2+^ imaging experiment, RFP-positive cells were visualized by exciting with a LED lamp at 530 nm. The fluorescence emission was collected at > 560 nm. In addition, a bright field image allowed visualization of all CD4^+^ T cells and thus calculation of the percentage of RFP-positive cells (FoxP3^+^ Treg). [Ca^2+^]_i_ was measured in single lymphocytes using excitation light provided by a LED lamp, with the beam passing through a monochromator at 340 nm and 380 nm with bandwidth of 10 nm (Cairn Research, Kent, UK). Emitted fluorescent light passed through a 515-nm long pass filter to a cooled CCD camera (Retiga; QImaging) and was digitized to 12-bit resolution. Ca^2+^ imaging data was acquired at a frame interval of 2 s and analyzed using MetaFluor^®^ Fluorescence Ratio Imaging Software (Molecular Devices, LLC). Traces were computed and plotted as fura-2 ratio of excitation acquired at 340 nm and 380 nm, both with emission at > 515 nm. Prior to stimulation of the cells with a mixture of 2 µg/mL biotin anti-CD3, 4 µg/mL biotin anti-CD28 and 40 µg/mL avidin, a 60 s baseline Ca^2+^ signal was recorded. The CD3/CD28-induced Ca^2+^ signal was measured in single cells due to the heterogeneous responses obtained. Ionomycin (1 µM) was given at the end of the experiment to assess viability of the cells. Ca^2+^ responses were visualized using Origin (OriginLab). A positive Ca^2+^ response was scored and measured if a fluorescence increase of 0.4 fura-ratio within 7.5 min after application of the CD3/CD28 mix was detected. This cutoff was defined to account for spontaneous non-CD3/CD28-driven Ca^2+^ oscillations which can be seen in lymphocytes. Areas under the curve (AUCs) were computed within a defined region using the AUC analyzer tool in Origin.

### Intracellular cytokine staining

For analysis of IL-10 production of Treg with flow cytometry, CD4^+^ T cells were first stimulated for 2 days with 2 µg/mL plate-bound anti-CD3 and 4 µg/mL soluble anti-CD28 in mouse T cell medium. Then cells were re-stimulated with 0.5 µl/mL leukocyte activation cocktail containing PMA, ionomycin and brefeldin A for 4 h. Cells were stained for CD3, CD4, CD8, CD25, FoxP3 and IL-10 using the FoxP3/Transcription factor staining buffer kit and analyzed by flow cytometry.

### Thymic organ cultures

P0 mice were sacrificed by decapitation and thymic lobes were removed, separated and transferred into 24-well Transwell^®^ dishes (pore size, 3 µm). The lower compartment of Transwell^®^ dishes was filled with 600 µL RPMI containing 10% FCS and 1% Pen/Strep. Pharmacological compounds (DMSO, CXQ and Lo) were added into the lower compartment and organ cultures were kept in the incubator (37 °C, 5% CO_2_) for 4–7 days. Thymic cell analysis was performed by homogenizing thymic lobes and/or collecting emigrated cells from the supernatant and subsequent flow cytometry analysis. T cell egress ratios were calculated as the absolute numbers of T cells that migrated into the lower compartment divided by the numbers of T cells found in the thymic lobes.

### TSDR methylation status

FACS-sorted Treg and Tconv from WT-*Foxp3*^*RFP*^ and *Kcnk18*^*G339R*^*/Foxp3*^*RFP*^ thymus and spleen were used for analysis of DNA methylation at the TSDR. Therefore, genomic DNA was isolated using Quick-DNA Microprep Plus Kit (Zymo Research) following the manufacturer’s instructions. Methylation analysis of the TSDR for low-input samples was performed by using bisulfite sequencing as described recently.^60^ We used the primers mTSDR-sen-for (5′-AGGTTGTTTTTGGGATATAGAATATG-3′), mTSDR-sen-rev (5′-ACCTATAAAATAAATTATCTACCCCCTTC-3′), mTSDR-sen-seq1 (5′-GTTGTTATAATTTGAATTTGGTTAG-3′) for amplification and pyrosequencing. The TSDR methylation analysis covers CpG motifs on chromosome X position 7450356–7450388 (genome assembly GRCm39).

### Transcriptomics

#### RNA isolation

FACS-sorted T cell subsets from thymus and spleen were used for analyzing the expression of *Kcnk18* by qPCR. RNA was isolated with Quick-RNA Microprep Kit (Zymo Research) following the manufacturer’s protocol. Tissue homogenates and cells were lysed in 300 µL RNA Lysis buffer, followed by sample clearing. The supernatant was mixed with 95%–100% ethanol and transferred to the column. In-column DNAse I treatment was performed. After washing and drying the column, RNA was eluted by pre-warmed DNase/RNase-free water (15 µL). RNA quality was assessed with NanoDrop by A260/A280 and A260/A230 ratios.

#### Real-time qPCR

Reverse transcription was performed with Maxima Reverse Transcriptase (Thermo Fisher Scientific) and random hexamer primers. 50 ng cDNA was used for Real-time qPCR with SYBR Green 2× Master Mix (Thermo Fisher Scientific). Therefore, 1 µM of each primer (mouse samples: mKcnk18fwd_qPCR and mKcnk18rev_qPCR; human samples: Hs_KCNK18_1_SG QuantiTect Primer Assay, Qiagen) or 1 µM housekeeping primer for the respective control (mouse samples: 18s-fwd and 18s-rev; human samples: pbgd-fwd and pbgd-rev), 10 µL SYBR Green Master mix, and 50 ng cDNA were mixed. PCR was performed on a Step-One-Plus Real-Time PCR System (Applied Biosystems) with the following steps: hold 2 min 50 °C, initial denaturation 10 min 95 °C, amplification (50×) 10 s 95 °C—45 s 58 °C—1 min 72 °C. Data were analyzed with StepOne software (Applied Biosystems, v2.1) calculating δC_T_ values and n-fold expression.

#### RNA sequencing and analysis

FACS-sorted tTreg from WT-*Foxp3*^*RFP*^ and *Kcnk18*^*G339R*^*/Foxp3*^*RFP*^ thymus were used for bulk RNA sequencing. RNA was isolated with Quick-RNA Microprep Kit (Zymo Research) as described above. Quality and amount of RNA were verified by NanoDrop and Bioanalyzer RNA 6000 Nano Kit (Agilent). Samples with RIN values > 6.5 were used for RNA sequencing. NEBNext^®^ rRNA depletion was performed followed by NEBNext directional Ultra RNA II Library preparation and sequencing on NextSeq500 (Illumina) platform (75 cycles, high output v2 kit). Raw sequencing data were analyzed by Linux bash tools following the analysis pipeline: (1) quality control (fastqc), (2) trimming (*Trimmomatic* 0.36),^61^ (3) alignment to mouse genome (*Hisat* 2.1.0; build: mm10),^62^ (4) aligned read sorting (*Samtools*)^63^ and (5) read counting (*HTseq* 0.10.0.^64^) Expression analysis was performed with R/Bioconducter *DESeq2.*^65^ Treg signature genes were assigned according to a previous study.^66^ Significantly regulated genes (FDR < 0.05) were used for further analysis and visualization using pHeatmap (https://cran.r-project.org/package=pheatmap) package in R. GO term gene enrichment analysis was performed using the PANTHER classification system online tool.^67,68^

### TCR sequencing

RNA from FACS-sorted Treg from spleen and lymph node was isolated using Quick-RNA Microprep Kit (Zymo Research) as described above and reverse transcribed into cDNA. The TCRβ chain was then amplified by multiplex PCR using primers specific for all 54 known expressed Vβ and all 13 Jβ regions and then subjected to deep sequencing by ImmunoSEQ^™^ (Adaptive Biotechnologies Corp., Seattle, WA, USA). The data were analyzed using the ImmunoSEQ™ Analyzer software and measures of diversity were evaluated. Shannon entropy is calculated by summing the frequency of each clone times the log_2_ of the same frequency over all productive reads in a sample. When this value is normalized based on the total number of productive unique sequences and subtracted from 1, clonality results. Values for clonality range from 0 to 1. Values near 1 represent samples with one or a few predominant clones (monoclonal or oligoclonal samples) dominating the observed repertoire. Clonality values near 0 represent more polyclonal samples.

### Analysis of transcription factor binding sites

2 kb upstream sequence including 5′UTR of mouse *Kcnk18* (Entrez Gene ID: 332396) was used for analysis of potential transcription factor binding sites. Binding site analysis was performed using ConSite (http://consite.genereg.net/).^69^ Putative transcription factors were reviewed manually in the context of Treg development. Sequences of binding sites were checked by browsing transcription factors with Jaspar (http://jaspar.genereg.net/) and compared to the predicted site in the 2 kb upstream/5′UTR sequence (Supplementary information, Table S1).

### Kinase activity profiliing

For serine-threonine kinase activity profiling, 6 biological replicates were sorted of both FoxP3^lo^ TregP and CD25^+^ TregP from WT-*FoxP3*^*RFP*^ and *Kcnk18*^*G339R*^*/FoxP3*^*RFP*^ thymus. Cells were lysed on ice for 15 min by homogenizing with M-PER Mammalian Protein Extraction Reagent supplemented with 1× Halt Protease and 1× Phosphotase Inhibitor Cocktail (Thermo Fisher Scientific). Afterwards, lysates were centrifuged for 15 min at 16,000× *g* and 4 °C. The supernatants were snap frozen in liquid nitrogen and stored at −80 °C until further processing. On the next day, kinase assays were performed using the serine-threonine kinase (STK) PamChip^®^ microarray system according to the manufacturer’s protocol (PamGene, HH’s-Hertogenbosch, The Netherlands). This multiplex phosphopeptide array-based methodology contains four positive controls and 140 serine/threonine peptides allowing the kinome-wide identification of hyper-activated kinases. Each of these peptides consists of a sequence of 15 amino acids which corresponds to a putative phosphorylation site serving as serine-threonine kinase substrates. As previously described,^70,71^ visualization of the phosphorylation activity of serine-threonine kinases was performed by a four-step protocol: blocking with 2% BSA (cycles 1–30) is followed by incubation of the protein lysates with a mixture of ATP and primary antibodies (assay mix, cycles 30–90). Next, samples are incubated with a fluorescently (FITC)-labeled secondary antibody (detection mix) and images are recorded (cycles 90–124). As previously stated,^72^ development of the fluorescence signal was detected by Alexa488 fluorescence. Measurements were performed on a Pamstation12 from PamGene. Unlike the company’s recommendation and due to low cell amounts after sorting, we analyzed only 5000 FoxP3^lo^ TregP and 10,000 CD25^+^ TregP per array. Kinase activity profiling and data analysis were performed using Evolve software and BioNavigator Analysis tool (PamGene). Signal intensities were analyzed as a function of time. The Upstream Kinase tool was used to identify differentially active kinases between those in *Kcnk18*^*G339R*^ and WT cells. The Kinase statistic (mean) score is calculated as the mean “treatment-control” group differences ((LFC)/Standard deviation), for a set of peptides assigned to a given kinase, derived from 6 current databases. The ranking factor is the Final (Median) Kinase Score (meanKS) which is based on a combined specificity score (derived from permutation of a set of peptides for a kinase) and significance score (derived from permutation of samples, in *Kcnk18*^*G339R*^ and WT groups).

### Immunofluorescence staining

#### Mouse thymus

Mouse thymus slices (10 µm) were fixed with 4% paraformaldehyde (PFA) for 10 min at 4 °C, washed three times with PBS and permeabilized with Fixation/Permeabilization buffer (eBioscience) for 30 min at RT, followed by blocking with 10% goat serum in PBS for 1 h at RT and a second blocking step using Avidin/Biotin Blocking Kit (15 min Avidin D, 15 min Biotin Solution). Slices were incubated with primary antibody (anti-FoxP3 Biotin, 1:30 in Permeabilization buffer) overnight at 4 °C. After washing, secondary antibody (Streptavidin-AF488, 1:1000 in Permeabilization buffer) was added to the slices and incubated for 3 h at RT. For CD4 staining, slices were washed, incubated first with anti-CD4 antibody (1:100 in PBS + 5% BSA for 2 h at RT) and second with Cy3 anti-rat antibody (1:500 in PBS + 1% BSA), and mounted with ProLong™ gold antifade mountant with DAPI (Invitrogen). Imaging was performed on Zeiss fluorescence microscope; ImageJ was used for cell counting.

#### Human thymus

Human thymus tissue of immunologically healthy patients was removed during necessary cardiac surgery (partial thymectomy) as a donation from patients for research proposes. The patient gave written informed consent according to national and European law, the declaration of Helsinki and the principles of Good clinical practice (GCP). All were approved by the faculty’s IRB (Ref# 46/00 and 45/00). For immunohistochemistry (IHC), tissue specimens were fixed in 4% PFA and embedded in paraffin. 4-µm-thick sections were used for IHC. IHC was performed using the Dako REALTM Detection System (Dako, #K5001), an automated immunostainer (AutostainerLink 48, Dako), and a biotin-streptavidin technique. Sections were deparaffinized and intrinsic peroxidase activity was blocked by incubation with 5% H_2_O_2_ in PBS for 5 min afterwards. We used mouse anti-FOXP3 (Abcam 20034; 1:100) and anti-TRESK (polyclonal, Immunoglobe; 1:100) as primary antibodies, and biotinylated secondary anti-mouse and -rabbit antibodies. For double-IHC, we used the abovementioned primary antibodies (1:50) followed by Cy3- or Alexa488-conjugated antibodies (1:250). Nuclei were stained using DAPI (1:5000, Invitrogen).

### Human subjects

All healthy individuals (*n* = 31, age: 18–52 years, average: 29.4 ± 0.8, 47% female, 53% male) and RRMS patients (*n* = 55, age: 17–54 years, 30.3 ± 1.7, 52% female, 48% male, diagnosed according to the McDonald criteria 2017)^73^ were recruited at the Departments of Neurology of the University Hospital Münster and the University Hospital Mainz in Germany. Patients received pre-treatments including β interferons and glatiramer acetate, teriflunomide, fingolimod and natalizumab.

For isolation of human RTE and nRTE populations of CD4^+^ Tconv and Treg, we used frozen PBMC samples from healthy individuals and patients with RRMS. PBMCs were stained for CD4, CD31, CD45RA, CD25, and CD127 and subsequently sorted by FACS isolating the following subsets: Tconv RTE (CD4^+^CD25^−^CD31^+^CD45RA^+^), Tconv nRTE (CD4^+^CD25^−^CD31^−^CD45RA^–^), Treg RTE (CD4^+^CD25^+^CD127^lo^ CD31^+^CD45RA^+^) and Treg nRTE (CD4^+^CD25^+^CD127^lo^ CD31^–^CD45RA^−^). For analysis of K_2P_18.1 expression on RTE and nRTE subsets using flow cytometry, cells were stained for CD3, CD4, CD25, CD31, CD45RA and K_2P_18.1. In some experiments female patients with uncomplicated UTI received nitroxoline (mean age: 33.5 years, 250 mg 3×/d) or nitrofurantoin (mean age: 31.25 years, 50 mg 4×/d) for 7 days according to the summary of product characteristics. PBMCs were isolated before, under (day 4 and day 6) and after treatment (day 14 and day 21).

For rs140325655 SNP analyses, genomic DNA was isolated from frozen PBMCs using the DNeasy Blood and Tissue Kit (Qiagen, Hilden, Germany) according to the manufacturer’s instructions. Genomic DNA was then hybridized to Infinium OmniExpress BeadChip arrays for whole-genome genotyping (Illumina, CA, USA). After processing and quality control of the genotyping data, phasing and imputation were performed using IMPUTE2 (version 2.3.2). Confirmation of SNP status was performed using the TaqMan® Genotyping assay (Assay ID: C_160407463_10; Thermo Fisher, CA, USA). Patients with confirmed major allele were compared to patients with heterozygosity for the minor allele and were matched for sex, age (±3 years) and first immunomodulatory treatment. For evaluation of clinically relevant disease-related outcomes (time from clinical disease onset to definite diagnosis of RRMS, cumulative baseline disability reflected by EDSS at baseline and in year two and annualized relapse rate).

The experiments were performed according to the declaration of Helsinki and approved by the local ethics committee (Ref# 2014-398-f-S). All healthy donors and RRMS patients gave written informed consent.

### Molecular biology and oocyte handling

hTRESK wt/pSGEM was a kind gift from Dr. Frank Döring (University of Wuerzburg). cDNA was linearized using *Nhe*I and in vitro transcription was performed using mMessage mMaschine T7 Transcription Kit (Invitrogen, Carlsbad, CA, USA). For two-electrode voltage clamp (TEVC) measurements, defolliculated oocytes were purchased from Ecocyte Bioscience (Dortmund, Germany). Oocytes were injected with 1 ng cRNA using Nanoliter Injector 2000 (WPI, Berlin, Germany) and stored at 18 °C in Barth solution for 48 h. Barth solution (containing 88 mmol/L NaCl, 1 mmol/L KCl, 0.4 mmol/L CaCl_2_, 0.33 mmol/L Ca(NO_3_)_2_, 0.6 mmol/L MgSO_4_, 5 mmol/L Tris-HCl, 2.4 mmol/L NaHCO_3_) was supplemented with 80 mg/L theophylline, 63 mg/L benzylpenicillin, 40 mg/L streptomycin and 100 mg/L gentamycin.

### Electrophysiology

Whole cell recordings from transfected HEK293 cells (48–72 h post transfection) and primary mouse tTreg were performed at RT in a bath solution consisting of 135 mM NaCl, 5.4 mM KCl, 1.8 mM CaCl_2_, 1 mM MgCl_2_, 10 mM glucose, and 5 mM HEPES, pH 7.4. Patch pipettes were pulled from borosilicate glass capillaries (Science Products, Hofheim, Gemany) and heat-polished to give input resistances of 3–6 megaohms. The pipette recording solution contained 140 mM KCl, 2 mM MgCl_2_, 1 mM EGTA, 1 mM Na_2_ATP, 100 μM cyclic AMP, and 5 mM HEPES, pH 7.3. Currents were recorded with an EPC9 (Heka) patch-clamp amplifier and low-pass filtered at 1–2 kHz. Stimulation and data acquisition were controlled by the Pulse/Pulsefit software package (Heka) on a Macintosh computer, and data analysis was performed with Igor software (WaveMetrics, Lake Oswego, OR).

In further experiments nitroxoline (Sigma-Aldrich) was prepared as 100 mM stock in DMSO and diluted with high-concentrated potassium buffer (containing 17.4 mmol/L NaCl, 80 mmol/L KCl, 1.8 mmol/L CaCl_2_ and 5 mmol/L HEPES (pH 7.4, adjusted using 1 M NaOH)) to a final concentration of 300 µM resulting in a DMSO concentration of 0.3%. Low-concentrated potassium buffer contained 95.4 mmol/L NaCl, 2 mmol/L KCl, 1.8 mmol/L CaCl_2_ and 5 mmol/L HEPES (pH 7.4, adjusted by 1 M NaOH). All test solutions were adjusted to a final DMSO concentration of 0.3%. Activity of nitroxoline in hTRESK-expressing *Xenopus laevis* oocytes was determined by TEVC at RT using previously described pulse protocols and recording pipettes (0.5–1.5 MΩ) backfilled with 3 M KCl.^74^ In short, oocytes were clamped to a holding potential of 0 mV and the following pulse protocol was applied 75 times (75 sweeps): 0 mV for 2 s, −100 mV for 0.5 s and 0 mV for 2 s. Low-potassium buffer (sweeps 1–15 and 61–75) followed by high-potassium buffer (sweeps 16–30 and 46–60) and 300 µM nitroxoline solution (sweeps 31–45) were sequentially superfused. Currents were normalized to the end of high-potassium buffer application (sweep 30) and effect of nitroxoline was evaluated at the end of compound application (sweep 45).

### Statistical analysis

For column-based data, Gaussian distribution was evaluated by D’Agostino-Pearson normality test. Dependent on normality for analysis of two groups, two-tailed *t*-test (unpaired/paired) or Mann–Whitney rank sum or Wilcoxon paired signed-rank test was used. If more groups were compared, we applied one way ANOVA, complemented by Bonferroni test for multiple comparisons for parametric data, or the Kruskal–Wallis test including Dunn’s post hoc test for non-parametric data. Linear regression analysis was performed using UNIANOVA following graphical inspection of residuals for presence of heteroskedasticity. For datasets with presence of this phenomenon, we used the heteroskedasticity-consistent 3 (HC3)-standard error estimates.^75^ Comparison of EAE data was performed using two way ANOVA. *P* values indicated were obtained from column-factor analysis (comparison between groups). Statistical analysis was performed using SPSS 27 (IBM, NY, USA) or GraphPad Prism. *P* > 0.05 were classified as not significant, *P* < 0.05 (*) as significant, *P* < 0.01 (**) and *P* < 0.001 (***) as highly significant.

## Supplementary information

Supplementary Figure 1

Supplementary Figure 2

Supplementary Figure 3

Supplementary Figure 4

Supplementary Figure 5

Supplementary Figure 6

Supplementary Figure 7

Supplementary Figure 8

Supplementary Figure 9

Supplementary Table 1

Glossary for abbreviations
