# Supplementary material for: K2P18.1 translates T cell receptor signals into thymic regulatory T cell development
Source: Cell Res. 2021 Oct 26;32(1):72–88. doi: 10.1038/s41422-021-00580-z (PMC8547300; doi:10.1038/s41422-021-00580-z)
Supplement: Supplementary file 1 — Supplementary Figure 1 [file 41422_2021_580_MOESM1_ESM.pdf]

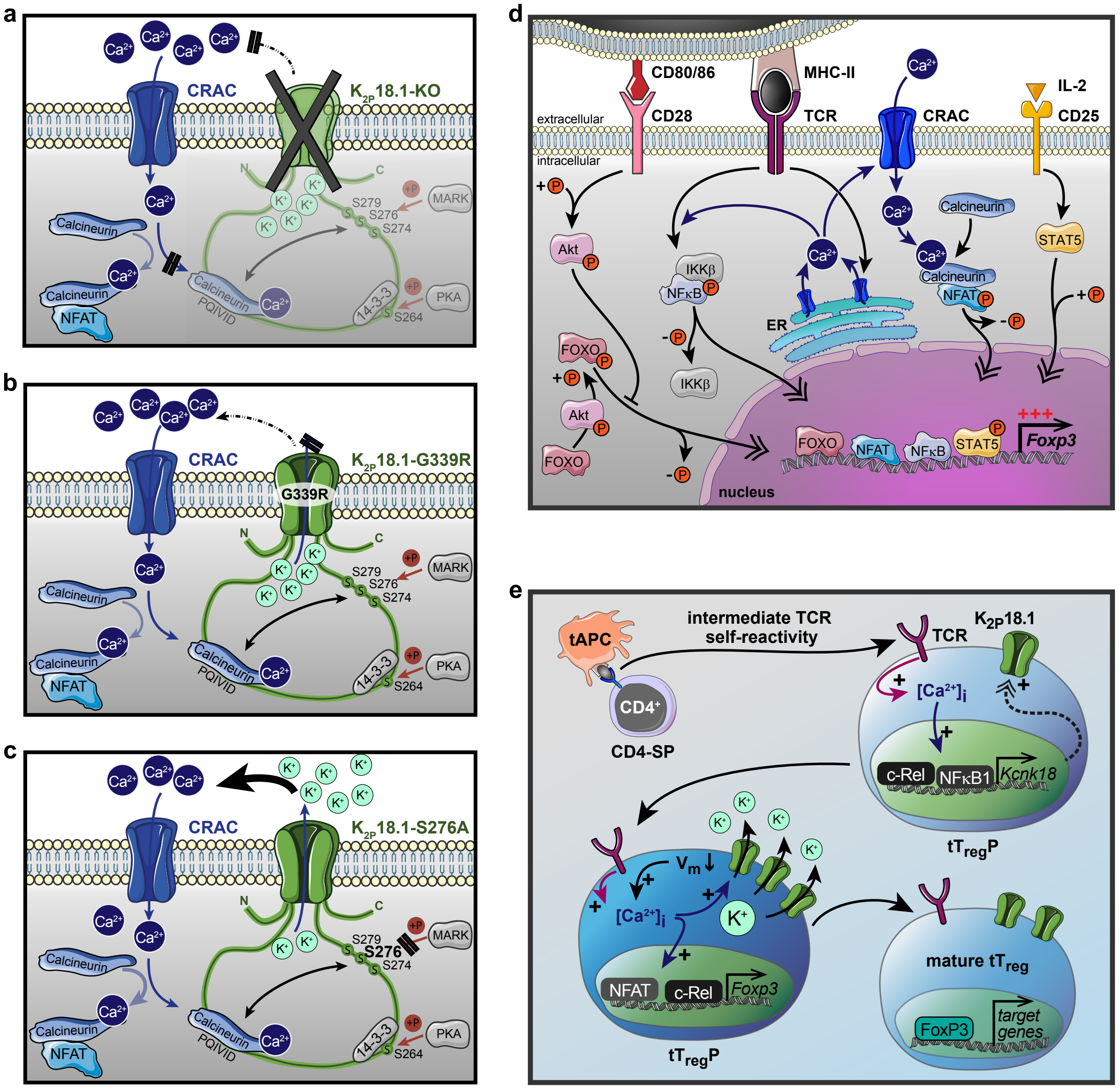

Supplementary figure 1 ***KCNK18 transgenic mouse models.*** **a** Scheme of $K_{2P}18.1$ function disrupted in full-knockout (*Kcnk18*$^{-/-}$) mice. Calcineurin binds to the NFAT-like PQIVID motif in the intracellular loop of $K_{2P}18.1$ and activates the channel by dephosphorylation of S-264 and the S-276 cluster. Phosphorylation of S-264 by PKA (protein kinase A) and S-276 by MARK (mammalian MAP/microtubule affinity-regulating kinase) restores the resting (inhibited) state of $K_{2P}18.1$. Docking of 14-3-3 adaptor protein to phosphorylated S-264 strengthens inhibition. **b** Scheme of G339R point mutation with abolished ion current through $K_{2P}18.1$ but intact intracellular signalling. **c** Scheme of S276A point mutation with facilitated ion current through $K_{2P}18.1$. **d** Scheme of T cell receptor (TCR) and IL-2 dependent intracellular signalling cascades leading to FoxP3 expression. **e** Scheme of $K_{2P}18.1$-mediated tT_{reg} development. T cell receptor (TCR) activation by antigens presented by thymic antigen-presenting cells (tAPC) drives fate decisions of multipotent CD4 single-positive thymocytes (CD4-SP) (step 1). TCR activation induces NF-κB signaling and thereby $K_{2P}18.1$ upregulation in tT_{reg} progenitors ($tT_{reg}P$) (step 2). $K_{2P}18.1$-mediated K^+ efflux hyperpolarizes the membrane potential (V_m) providing the driving force for prolonged high intracellular Ca^{2+} levels, which in turn activate $K_{2P}18.1$-mediated ion-conductance and NF-κB signaling forming a positive feedback loop. High intracellular Ca^{2+} levels further promote the translocation of NFAT and NF-κB related transcription factors into the nucleus (step 3). Those transcription factors induce and stabilize FoxP3 expression and thereby the development of mature tT_{reg} (step 4).
