# Supplementary material for: K2P18.1 translates T cell receptor signals into thymic regulatory T cell development
Source: Cell Res. 2021 Oct 26;32(1):72–88. doi: 10.1038/s41422-021-00580-z (PMC8547300; doi:10.1038/s41422-021-00580-z)
Supplement: Supplementary file 2 — Supplementary Figure 2 [file 41422_2021_580_MOESM2_ESM.pdf]

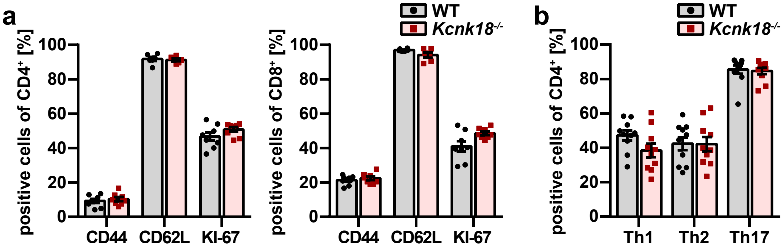


**Supplementary figure 2 Markers for T cell activation and *in vitro* Th cell induction show no differences in WT and *Kcnk18*$^{-/-}$ mice.** **a** Surface expression of indicated activation markers on $CD4^+$ and $CD8^+$ $T_{conv}$ isolated from WT and *Kcnk18*$^{-/-}$ mice. (n = 4-6). **b** *In vitro* Th1, Th2 and Th17 induction from naïve $CD4^+CD25^-CD44^-$ T cells in the presence of plate-bound anti-CD3 (2µg/ml), soluble anti-CD28 (4µg/ml) and different cytokines and antibodies driving Th cell differentiation. (n=10). Data are represented as mean ± SEM. * $p < 0.05$, ** $p < 0.01$, *** $p < 0.001$.
