# Supplementary figures and images for: K2P18.1 translates T cell receptor signals into thymic regulatory T cell development
Source: Cell Res. 2021 Oct 26;32(1):72–88. doi: 10.1038/s41422-021-00580-z (PMC8547300; doi:10.1038/s41422-021-00580-z)

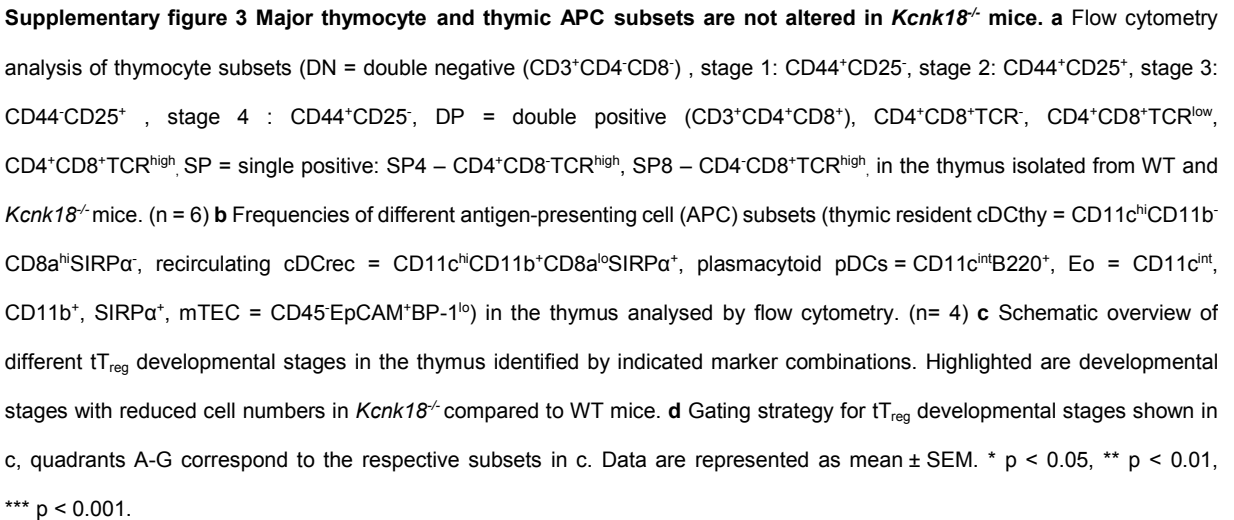

Supplement: Supplementary file 3 — Supplementary Figure 3 [file 41422_2021_580_MOESM3_ESM.pdf]
