# Supplementary material for: K2P18.1 translates T cell receptor signals into thymic regulatory T cell development
Source: Cell Res. 2021 Oct 26;32(1):72–88. doi: 10.1038/s41422-021-00580-z (PMC8547300; doi:10.1038/s41422-021-00580-z)
Supplement: Supplementary file 4 — Supplementary Figure 4 [file 41422_2021_580_MOESM4_ESM.pdf]

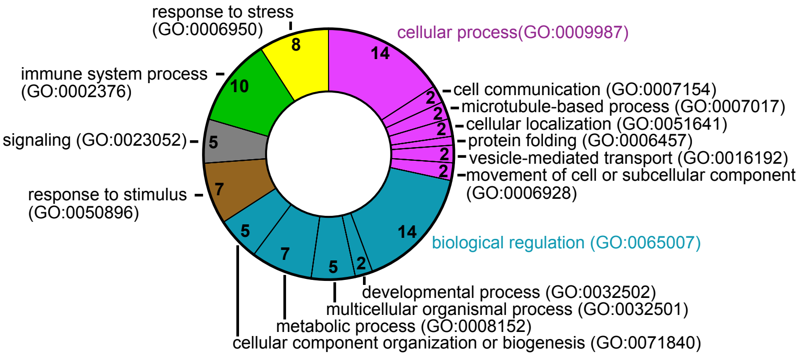

Supplementary figure 4 GO-Term gene enrichment analysis of significantly differentially regulated genes (FDR) in *Kcnk18*^{G339R} versus WT tT$_{reg}$. GO-Term gene enrichment analysis was performed using the PANTHER classification system. Numbers of differentially regulated genes assigned to the respective GO-Term are depicted within the pie-chart. The GO-Terms "cellular process (GO:0009987)" (purple) and "biological regulation (GO:0065007)" (blue) were further sub classified.
