# Supplementary material for: K2P18.1 translates T cell receptor signals into thymic regulatory T cell development
Source: Cell Res. 2021 Oct 26;32(1):72–88. doi: 10.1038/s41422-021-00580-z (PMC8547300; doi:10.1038/s41422-021-00580-z)
Supplement: Supplementary file 5 — Supplementary Figure 5 [file 41422_2021_580_MOESM5_ESM.pdf]

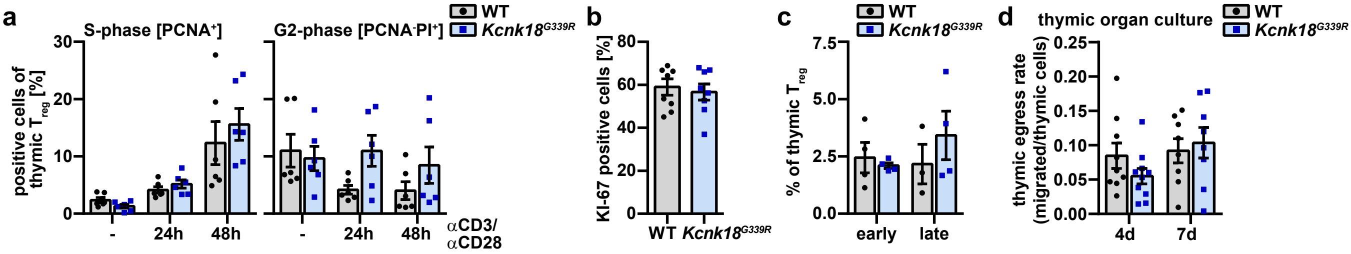

Supplementary figure 5 Cell cycle and apoptosis of tT_{reg} in *Kcnk18*^{G339R} compared to WT mice. a Cell cycle analysis in WT and *Kcnk18*^{G339R} tT_{reg} isolated from thymus. Frequencies of cells in S-($PCNA^{+}PI^{+/-}$) and G2-($PCNA^{-}PI^{+}$) phases were evaluated by flow cytometry. (n = 6) **b** Frequencies of early (Caspase3/$7^{+}FVD^{-}$) and late apoptotic (Caspase3/$7^{+}FVD^{+}$) tT_{reg} isolated from WT and *Kcnk18*^{G339R} thymus. (n = 6). **c** Proportion of $KI67^{+}$ tT_{reg} in thymus of WT and *Kcnk18*^{G339R}. (n = 8) **d** tT_{reg} egression rates (emigrated cells/thymic cells) in WT and *Kcnk18*^{G339R} thymus organ cultures after 4 and 7 days. (n = 8). Data are represented as mean ± SEM. * $p < 0.05$, ** $p < 0.01$, *** $p < 0.001$.
