# Supplementary material for: K2P18.1 translates T cell receptor signals into thymic regulatory T cell development
Source: Cell Res. 2021 Oct 26;32(1):72–88. doi: 10.1038/s41422-021-00580-z (PMC8547300; doi:10.1038/s41422-021-00580-z)
Supplement: Supplementary file 6 — Supplementary Figure 6 [file 41422_2021_580_MOESM6_ESM.pdf]

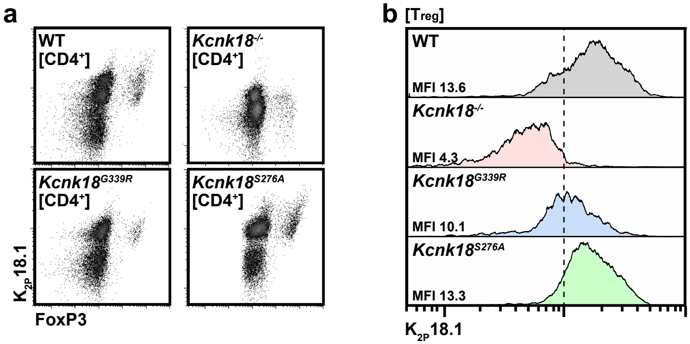

Supplementary figure 6 Flow cytometry analysis of K_{2P}18.1 expression. a Representative flow cytometry dot blots of K_{2P}18.1 and FoxP3 expression in WT, *Kcnk18*$^{-/-}$, *Kcnk18*^{G339R} and *Kcnk18*^{S276A} CD4-SP thymocytes. **b** Representative flow cytometry histogram of K_{2P}18.1 expression in WT (grey), *Kcnk18*$^{-/-}$ (red), *Kcnk18*^{G339R} (blue) and *Kcnk18*^{S276A} (green) tT_{reg}.
