# Supplementary material for: K2P18.1 translates T cell receptor signals into thymic regulatory T cell development
Source: Cell Res. 2021 Oct 26;32(1):72–88. doi: 10.1038/s41422-021-00580-z (PMC8547300; doi:10.1038/s41422-021-00580-z)
Supplement: Supplementary file 7 — Supplementary Figure 7 [file 41422_2021_580_MOESM7_ESM.pdf]

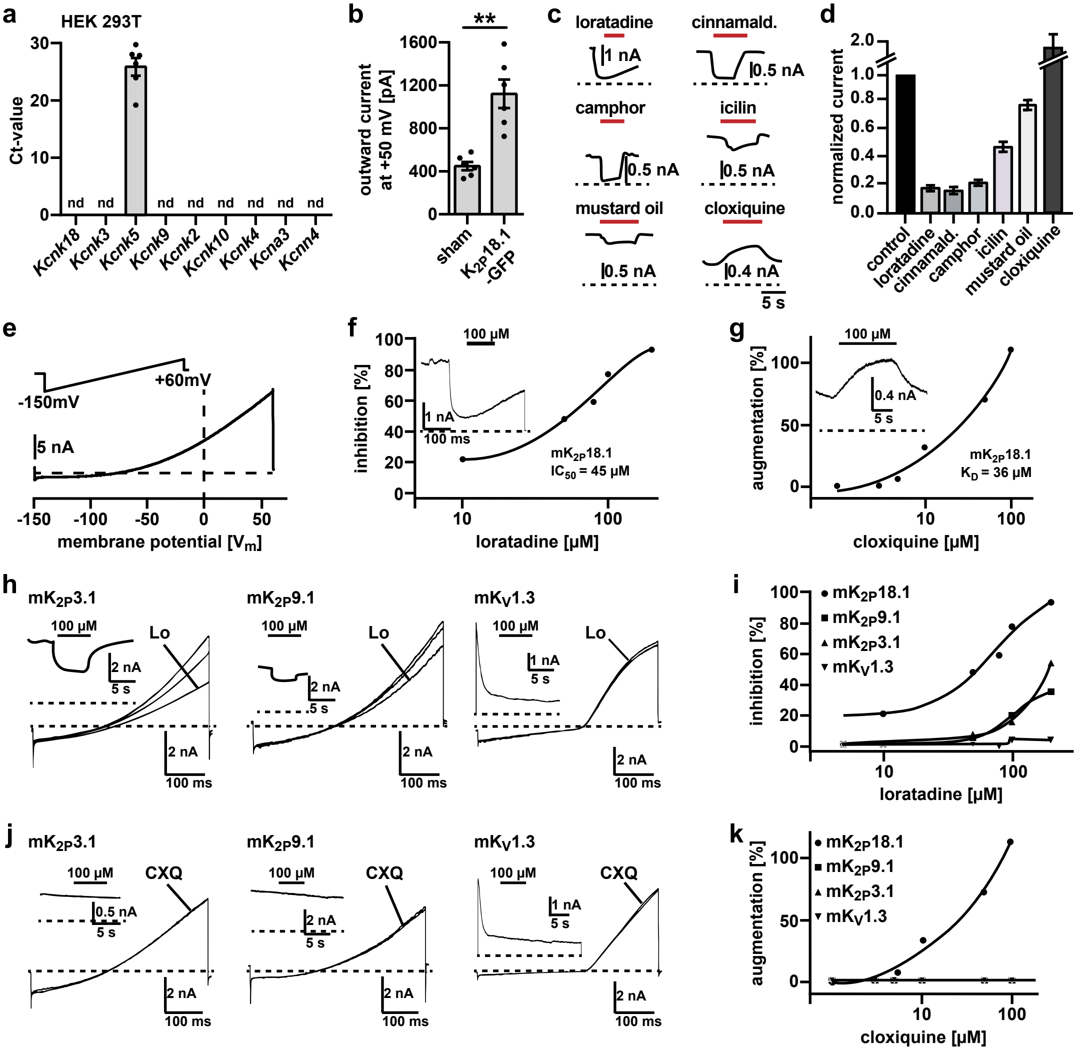

Supplementary figure 7 Electrophysiological characterization of K_{2P}18.1 pharmacological modulator. a qPCR expression profile of different potassium channels in non-transfected HEK 293T cells. (n = 6) **b** Whole cell patch clamp recordings of potassium outward currents in K_{2P}18.1-GFP transfected HEK 293T cells at a membrane potential of +50 mV. (n = 6) **c** Representative recordings of K_{2P}18.1 current measured at +35 mV after application of different modulators. **d** Quantification of maximal inhibition of modulators from (**c**) inhibition: loratadine (100 µM, n = 6), cinammaldehyde (3 mM, n = 6), camphor (10 mM, n = 5), icilin (50 µM, n = 9) and mustard oil (10 mM, n = 10); activation: cloxiquine (100 µM, n = 6). **e** Voltage ramp protocols from -150 mV to +60 mV were used to evoke potassium outward currents. **f** Dose response curve of inhibition in K_{2P}18.1-GFP transfected HEK 293T cells treated with increasing concentrations of loratadine as indicated, IC_{50} = concentration of 50% maximal inhibition. (n = 3, each concentration) **g** Dose response curve of activation in K_{2P}18.1-GFP transfected HEK 293T cells treated with increasing concentrations of cloxiquine as indicated, (n = 3, each concentration). Ramp recordings of outward currents from transfected HEK 293T cells with the indicated potassium channels after application of different loratadine and cloxiquine concentrations as indicated: Representative current traces with application of 50 µM and 100 µM loratadine **h** or cloxiquine **j** with the respective dose response curves **i** + **k** (n = 3 each). Data are represented as mean ± SEM. * $p < 0.05$, ** $p < 0.01$, *** $p < 0.001$.
