# Supplementary material for: K2P18.1 translates T cell receptor signals into thymic regulatory T cell development
Source: Cell Res. 2021 Oct 26;32(1):72–88. doi: 10.1038/s41422-021-00580-z (PMC8547300; doi:10.1038/s41422-021-00580-z)
Supplement: Supplementary file 8 — Supplementary Figure 8 [file 41422_2021_580_MOESM8_ESM.pdf]

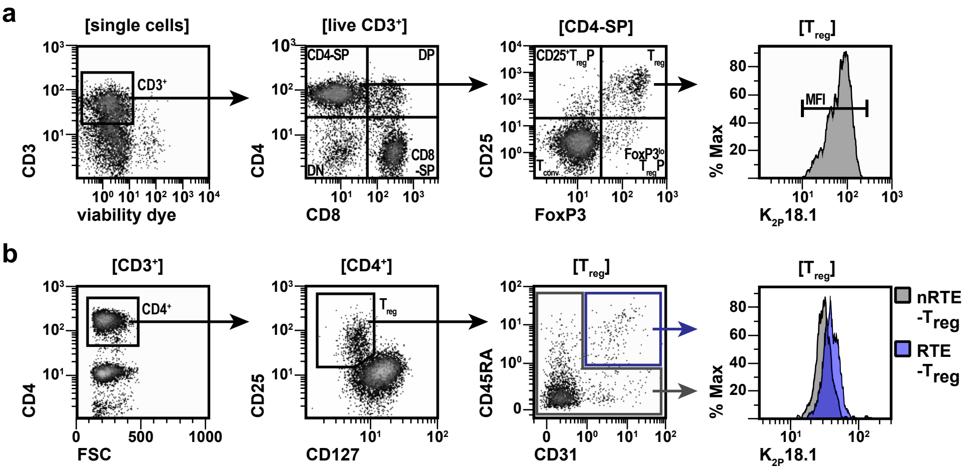

Supplementary figure 8 Flow cytometry gating strategy a Representative gating of mouse T_{reg} ($CD3^+CD4^+CD8^-CD25^+FoxP3^+$) in WT thymocytes. Single cells were used for gating on live $CD3^+$ T cells, followed by discrimination of $CD4^+$ and $CD8^+$. CD4-SP were further gated for CD25 and FoxP3 to identify thymic $CD4_{conv}$, T_{reg} and $T_{reg}P$. Expression levels of $K_{2P}18.1$ has been assessed by measurements of the mean fluorescence intensity in the cell population of interest. **b** Gating of human RTE T_{reg} ($CD4^+CD45RA^+CD31^+CD25^+CD127^{lo}$) and non-RTE T_{reg} ($CD4^+CD45RA^-CD31^+CD25^+CD127^{lo}$, $CD4^+CD45RA^+CD31^-CD25^+CD127^{lo}$, $CD4^+CD45RA^-CD31^-CD25^+CD127^{lo}$).
