# Supplementary material for: K2P18.1 translates T cell receptor signals into thymic regulatory T cell development
Source: Cell Res. 2021 Oct 26;32(1):72–88. doi: 10.1038/s41422-021-00580-z (PMC8547300; doi:10.1038/s41422-021-00580-z)
Supplement: Supplementary file 9 — Supplementary Figure 9 [file 41422_2021_580_MOESM9_ESM.pdf]

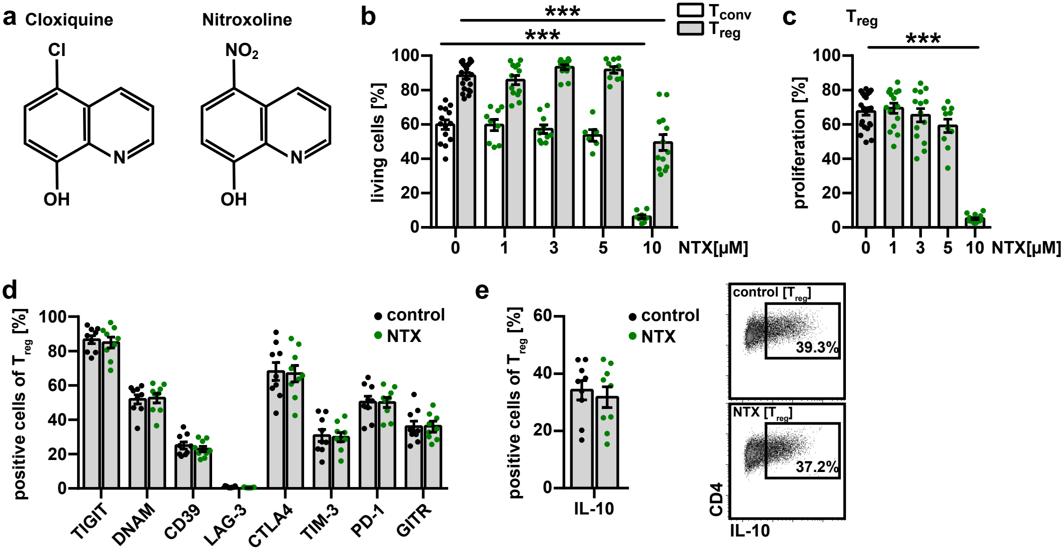

Supplementary figure 9 Proliferation and function of human T_{reg} under nitroxoline treatment *in vitro.* a Chemical structure of cloxiquine and nitroxoline. **b** Quantification of living T cells under nitroxoline (NTX) treatment. $CD4^+$ T cells were isolated from healthy controls and treated with plate-bound anti-CD3 (1µg/ml), soluble anti-CD28 (2µg/ml) and the indicated NTX concentrations for 7d. Cells were then stained with AnnexinV and FVD and living T_{conv} and T_{reg} cells were quantified by flow cytometry as $AnnexinV^-FVD^-$ cells. (n=14) **c** CFSE proliferation assay of sorted human T_{reg} ($CD4^+CD25^{hi}CD127^{lo}$) isolated from healthy controls. T_{reg} were stimulated with plate-bound anti-CD3 (1µg/ml), soluble anti-CD28 (2µg/ml) and indicated concentrations of nitroxoline (NTX) for 7d and analysed by flow cytometry. (n = 14) **d** Surface expression of indicated effector molecules on T_{reg} isolated from healthy controls and stimulated with plate-bound anti-CD3 (1µg/ml), soluble anti-CD28 (2µg/ml) and xx µM nitroxoline (NTX) for 7d. (n = 9). **e** Intracellular cytokine staining for IL-10: MACS-isolated $CD4^+$ T cells were stimulated with plate-bound anti-CD3 (2.0 µg/ml) and soluble anti-CD28 (4.0 µg/ml) for 24h, then re-stimulated with leukocyte activation cocktail (LAC (0.5 µl/ml), containing PMA, ionomycin and Brefeldin A) for 4h and analysed by flow cytometry. Left: bar graphs, right: representative dot plots. (n = 9). Data are represented as mean ± SEM. * $p < 0.05$, ** $p < 0.01$, *** $p < 0.001$
