# Supplementary material for: K2P18.1 translates T cell receptor signals into thymic regulatory T cell development
Source: Cell Res. 2021 Oct 26;32(1):72–88. doi: 10.1038/s41422-021-00580-z (PMC8547300; doi:10.1038/s41422-021-00580-z)
Supplement: Supplementary file 10 — Supplementary Table 1 [file 41422_2021_580_MOESM10_ESM.pdf]

Supplementary table 1 Putative transcription factor binding sites in the *Kcnk18* promoter region. *Kcnk18* Entrez gene ID:332396 was used for transcription factor binding site analysis with ConSite.[75] Regions correspond to the 2 kb upstream sequence and 5`UTR. The indicated score depicts the sequence similarity to known transcription factor binding sites in JASPAR database.

Transcription Factor	Sequence	from-to	score [strand]
**c-Fos / Jun**	GTGATTAA	470-477	9.471 [+]
**c-Fos / Jun**	GTGATTAA	941-948	10.264 [+]
**n-Myc**	CACGTG	1241-1246	10.533 [+]
**n-Myc**	CACGTG	1550-1555	10.351 [+]
**p65 /c-Rel**	CAGAATTTCC	1612-1621	9.927 [+] / 7.686 [+]
**p65**	CAGAGTTTCC	1642-1651	8.799 [+]
**p65 /c-Rel**	AGGGGTTTCC	1975-1984	10.053 [+] / 9.028 [+]
**NF-kappaB**	GGGGTTTCCA	1976-1989	7.776 [+]
**NF-kappaB**	GGCAAGTTCC	2008-2017	7.763 [+]
**RORalfa-1**	TTCCAGGTCA	2014-2023	11.473 [+]
**IRF-1**	GAAACCGAAGCT	2190-2201	10.277 [+]
