# Supplementary material for: K2P18.1 translates T cell receptor signals into thymic regulatory T cell development
Source: Cell Res. 2021 Oct 26;32(1):72–88. doi: 10.1038/s41422-021-00580-z (PMC8547300; doi:10.1038/s41422-021-00580-z)
Supplement: Supplementary file 11 — Glossary for abbreviations [file 41422_2021_580_MOESM11_ESM.pdf]

Supplementary Material and Methods

Software for Data acquisition and analysis

Kaluza v1.1 for Gallios (Beckman-Coulter), FACS Diva Software v8.0 (BD Bioscience), StepOne Software v2.1 (Applied Biosystems), MetaFluor (Molecular Devices), Pulse/Pulsefit software package (Heka), AMNIS INSPIRE (Luminex), ImmunoSEQ™ (Adaptive Biotechnologies Corp.), Kaluza Analysis v2.1 (Beckmann-Coulter), StepOne Software v2.1 (Applied Biosystems), AMNIS IDEA (Luminex), ImageJ, R Studio v3.5.2: R package pheatmap v1.0.12, R package tidyverse v1.2.1, Trimmomatic v0.36, Hisat v2.1.0, Samtools, HTseq v0.10.0, DESeq2, Origin (Additive Origin), ImmunoSEQ™ Analyzer software (Adaptive Biotechnologies), Igor (Wave Metrics), YASARA-Structure v17.12.24 (YASARA Biosciences GmbH), Evolve (PamGene), BioNavigator Analysis tool (PamGene).

Antibodies (clone)

APC anti-mouse/rat FOXP3 Antibody (FJK-16s), eBioscience #17-5773-82; Biotin anti-mouse/rat FOXP3 Antibody (FJK-16s), eBioscience #13-5773-82; Anti-human FOXP3 Antibody (236A/E7), Abcam #ab20034; Pacific Blue™ anti-mouse CD4 Antibody (GK1.5), BioLegend #100428; Brilliant Violet 510™ anti-mouse CD3 Antibody (17A2), BioLegend #100234; PerCP/Cyanine5.5 anti-mouse CD3 Antibody (17A2), BioLegend #100218; PE/Dazzle™ 594 anti-mouse CD3 Antibody (17A2), BioLegend #100246; PE/Cy7 anti-mouse CD25 Antibody (PC61), BioLegend #102016; FITC anti-mouse/human Helios Antibody (22F6), BioLegend #137214; Alexa Fluor® 700 anti-mouse CD8a Antibody (53-6.7), BioLegend #100730; Brilliant Violet 605™ anti-mouse CD8a Antibody (53-6.7), BioLegend #100744; Brilliant Violet 510™ anti-mouse CD8a Antibody (53-6.7), BioLegend #100752; PE anti-mouse/rat FOXP3 Monoclonal Antibody (FJK-16s), eBioscience #12-5773-82; PE anti-mouse CD357 (GITR) Antibody (DTA-1), BioLegend #126310; PerCP/Cyanine5.5 anti-mouse CD73 Antibody (TY/11.8), BioLegend #127214; PE anti-mouse Nur77 Antibody (12.14), eBioscience #12-596482; Alexa Fluor® 488 anti-human/mouse/rat PCNA Antibody (PC10), BioLegend #307909; Brilliant Violet 421™ anti-mouse/human Ki-67 Antibody (11F6), BioLegend #151208;

Purified anti-mouse CD28 Antibody (37.51), BioLegend #102102; Biotin anti-mouse CD28 Antibody (37.51), BioLegend #102104; Biotin anti-mouse CD3ε Antibody (145-2C11), BioLegend #100304; Purified anti-mouse CD3ε Antibody (145-2C11), BioLegend #100302; Purified anti-mouse IL-4 Antibody (11B11), BioLegend #504102; Purified anti-mouse IFN-γ Antibody (AN-18), BioLegend #517902; PE anti-mouse/human CD45R/B220 Antibody (RA3-6B2), BioLegend #103207; Alexa Fluor® 700 anti-mouse CD19 Antibody (6D5), BioLegend #115527; APC anti-mouse NK-1.1 Antibody (PK136), BioLegend #108709; FITC anti-mouse CD11c Antibody (N418), BioLegend #117305; Brilliant Violet 510™ anti-mouse/human CD11b Antibody (M1/70), BioLegend #101245; PE anti-mouse IL-10 Antibody (JES5-16E3), BioLegend #112705; APC anti-mouse TCR β chain Antibody (H57-597), BioLegend #109211; PE anti-mouse CD278 (ICOS) Antibody (7E.17G9), BD PharmingenTM #552146; PerCP/Cyanine5.5 anti-mouse CD69 Antibody (H1.2F3), BioLegend #104522; PE anti-mouse CD5 Antibody (53-7.3), BioLegend #100607; FITC anti-mouse/human CD44 Antibody (IM7), BioLegend #103021; APC anti-mouse CD326 (Ep-CAM) Antibody (G8.8), BioLegend #118213; PE/Cy7 anti-mouse CD86 Antibody (GL-1), BioLegend #105013; APC anti-mouse CD172a (SIRPα) Antibody (P84), BioLegend #144013; APC-eFluor780 anti-mouse MHC Class II (I-A/I-E) Antibody (M5/114.15.2), eBioscience #47-5321-82; PE anti-mouse CD45 Antibody (30-F11), BioLegend #103106 ; NF-kB p65 XP® anti-mouse monoclonal Antibody (D14E12), Cell Signaling #8242; NFAT1 XP® anti-mouse monoclonal Antibody, Cell Signaling #5861; Phospho-Akt (Ser473) anti-mouse monoclonal Antibody (193H12), Cell Signaling #4058; Phospho-Stat5 (Tyr694) anti-mouse/human Antibody, Cell Signaling #9351; TRESK anti-mouse monoclonal Antibody (E-2), Santa Cruz #sc-514525; Anti-human TRESK Antibody, ImmunoGlobe N/A; Alexa Fluor® 488 goat anti-mouse IgG H&L, abcam #ab150113; APC donkey anti-rabbit IgG H&L, Jackson Immuno Research (Dianova) #711-136-152; FITC anti-human CD45RA (HI100), BioLegend #983002; PE/Cy7 anti-human CD31 Antibody (WM59), BioLegend #303118; Brilliant Violet 510™ anti-human CD127 (IL-7Rα) Antibody (A019D5), BioLegend #351331; Brilliant Violet 421™ anti-human CD4 Antibody (OKT4), BioLegend #317434; APC anti-human CD25 Antibody (BC96), BioLegend #302610; Purified anti-mouse

CD4 Antibody (L3T4, T4), BioLegend #100506; AF488 goat anti-mouse IgG H&L, Invitrogen #A28175; CyTM3 donkey anti-rat IgG H&L, Jackson Immuno Research (Dianova) #712-166-153; APC donkey anti-rabbit IgG H&L, Jackson Immuno Research (Dianova) #711-136-152; PE anti-human FoxP3 (PCH101), eBioscience #12-4776-42; PerCp-Cy5.5 anti-human CD31 (WM59), Biolegend #303132; PE-Cy7 anti-human TIGIT (MBSA43), eBioscience #25-9500-42; APC anti-human CD226/DNAM1 (11A8), Biolegend #338312; APC-A700 anti-human CD127 (R34.34) Beckman Coulter #A7116; APC-A750 anti-human CD3 (UCHT1) Beckman-Coulter #A94680; BV421 anti-human CD39 (A1), Biolegend #328214; BV510 anti-human CD8 (SK1), Biolegend #344732; BV650 anti-human Cd25 (Bc96), Biolegend #302634; BV785 anti-human CD4 (OKT4), Biolegend #317442; FITC anti-human CD223/LAG3 (7H2C65), Biolegend #369210; PE-Cy7 anti-human CD152/CTLA4 (BNI3), Biolegend #369614; APC anti-human TIM3 (F38-2E2), Miltenyi Biotec #130-098-936; APC-Cy7 anti-human Cd8 (HIT8a), Biolegend #300926; BV421 anti-human CD279/PD-1 (EH12.2H7), Biolegend # 329919; BV650 anti-human CD3 (UCHT1), Biolegend #300468; BV785 anti-human CD4 (OKT4), Biolegend #317442; APC anti-mouse TCR Vβ11 (KT11), Biolegend #125914; Alexa Fluor® 700 anti-mouse/human CD11b (M1/70), Biolegend #101222; Brilliant Violet 650™ anti-mouse CD3 (17A2), Biolegend #100229; FITC anti-mouse IFN-γ (XMG1.2), Biolegend #505806; PE anti-mouse IL-4 (TC11-18H10.1), Biolegend #506916; APC/Cyanine7 anti-mouse CD45 (30-F11), Biolegend #103116; FITC anti-mouse CD279 (PD-1) (29F.1A12), Biolegend #135214; PE anti-mouse CD152 (UC10-4B9), Biolegend # 106306; PE/Dazzle™ 594 anti-mouse Ki-67 (16A8), Biolegend #652428; APC/Cyanine7 anti-mouse CD62L (MEL-14), Biolegend # 104427; FITC anti-mouse GITR (DTA-1), Biolegend #126308; PE anti-mouse TIGIT (Vstm3) (4D4/mTIGIT), Biolegend #156103; Alexa Fluor® 700 anti-human/mouse/rat CD278 (ICOS) (C398.4A), Biolegend #313528; FITC anti-mouse/human CD44 (IM7), Biolegend #103006; Alexa Fluor® 700 anti-mouse/human Helios (22F6), Biolegend #137242; PE anti-mouse IL-10 (JES5-16E3), Biolegend #505008; Brilliant Violet 421™ anti-human CD45RA (HI100), Biolegend #304129; Brilliant Violet 605™ anti-human CD4 (OKT4), Biolegend #317437; Brilliant Violet 650™ anti-human CD25 (BC96), Biolegend #302633; PerCP/Cyanine5.5 anti-

human Ki-67 (Ki-67), Biolegend #350519; APC anti-human IL-10 (JES3-19F1), Biolegend #506806; Alexa Fluor 700 anti-mouse CD39 (24DMS1), eBioscience #56-0391-82.

Glossary for abbreviations

DN	CD4$^-$CD8$^-$ double negative CD3$^+$ thymocyte
DP	CD4$^+$CD8$^+$ double positive CD3$^+$ thymocyte
SP	CD4$^+$CD8$^-$/ CD4$^-$CD8$^+$ single positive CD3$^+$ thymocyte
Th cell	T helper cell
T_{conv}	CD3$^+$CD8$^-$CD4$^+$CD25$^-$FoxP3$^-$ conventional T cell
T_{reg}	regulatory T cell
pT_{reg} / tT_{reg} / iT_{reg}	peripherally-induced / thymus-derived / *in vitro* induced T_{reg}
T_{reg}P	T_{reg} progenitor cell
mTEC	thymic medullary epithelial cells
RTE	recent thymic emigrants (CD45RA$^+$CD31$^+$)
DRG	dorsal root ganglia
WT	wildtype mice
KO	*Kcnk18*$^{-/-}$, full knock-out of K_{2P}18.1
GR	*Kcnk18*^{G339R}, functional knock-out of K_{2P}18.1 (inhibition of K_{2P}18.1-mediated K^+ current)
SA	*Kcnk18*^{S276A}, K_{2P}18.1 gain of function mutation (enhanced K_{2P}18.1-mediated K^+ current)
TCR	T cell receptor
TSDR	T_{reg} specific demethylated region
UTR	untranslated region
CXQ	cloxiquin, K_{2P}18.1 activator
Lo	loratadine, K_{2P}18.1 inhibitor
CDN	cardamonin, NF-kB translocation inhibitor
PTN	parthenolide, NF-kB translocation inhibitor
NTX	nitroxoline
EAE	experimental autoimmune encephalomyelitis
(RR)MS	(relapsing-remitting) multiple sclerosis
